# Recent developments on triazole nucleus in anticonvulsant compounds: a review

**DOI:** 10.1080/14756366.2017.1423068

**Published:** 2018-01-31

**Authors:** Ming-Xia Song, Xian-Qing Deng

**Affiliations:** Medical College, Jinggangshan University, Ji’an, Jiangxi, China

**Keywords:** Epilepsy, anticonvulsant, triazole, MES, scPTZ

## Abstract

Epilepsy is one of the common diseases seriously threatening life and health of human. More than 50 million people are suffering from this condition and anticonvulsant agents are the main treatment. However, side effects and intolerance, and a lack of efficacy limit the application of the current anticonvulsant agents. The search for new anticonvulsant agents with higher efficacy and lower toxicity continues to be the focus and task in medicinal chemistry. Numbers of triazole derivatives as clinical drugs or candidates have been frequently employed for the treatment of various types of diseases, which have proved the importance of this heterocyclic nucleus in drug design and discovery. Recently many endeavours were made to involve the triazole into the anticonvulsants design, which have brought lots of active compounds. This work is an attempt to systematically review the research of triazole derivatives in the design and development of anticonvulsant agents during the past two decades.

## Introduction

Epilepsy, a group of neurological disorders characterised by epileptic seizures, afflict over 50 million people around the world^1^^,^^2^. The cause of most cases of epilepsy is unknown^3^. Some cases occur as the result of brain injury, stroke, brain tumours, infections of the brain, and birth defects, through a process known as epileptogenesis^3–5^. Known genetic mutations are directly linked to a small proportion of cases^6^^,^^7^. The acceptable pathogenesis of epileptic seizures is the imbalance of excitatory and inhibitory neurotransmitters in central nervous system, which lead an abnormal nerve cell activity and neuronal discharge resulting in seizures^4^. Because the exact mechanism of epilepsy is unknown, there is still no medical cure for epilepsy (epileptogenesis)^8^^,^^9^. The anticonvulsants (also commonly known as antiepileptic drugs or antiseizure drugs), “symptomatic” agents that suppress the symptoms of epilepsy (i.e. seizures), was the main strategy for epilepsy treatment.

Since the discovery of the first anticonvulsant bromide in 1857, a large number of anticonvulsants were developed and approved for epilepsy treatment: phenobarbital, phenytoin, primidone, methsuximide, methazolamide, ethotoin, diazepam, trimethadione, sodium valproate, clonazepam, clobazam, carbamazepine, acetazolamide, valproic acid, felbamate, fosphenytoin, gabapentin, lamotrigine, lacosamide, levetiracetam, oxcarbazepine, stiripentol, vigabatrin, zonisamide, rufinamide, retigabine, and so on^10–12^. For patients with epilepsy, a single medication is recommended initially^13^. But there are about half of seizures could not be controlled by using a single medication (monotherapy), then polytherapy with multiple anticonvulsants is recommended^14^. Unfortunately, about 30% of people continue to have seizures despite anticonvulsants treatment^15^, and the side effects of anticonvulsant agents follow up. Until now, the existing drugs are far from ideal, being consistently effective in fewer than 70% of patients and tending to produce a variety of side-effects in more than 50% of patients^8^. Toxicity, intolerance, and a lack of efficacy represent the limitations of the available anticonvulsants, which stimulated the continual attempts for discovery of new anticonvulsants.

Since 1975, the Epilepsy Branch of the National Institute of Neurological Disorders and Stroke, National Institutes of Health, through its Antiepileptic Drug Development (ADD) Program, has collaborated with the pharmaceutical industry and academic individual in developing new therapeutic agents for the treatment of seizure disorders^16^. The program has made important contributions to the development of several FDA-approved drugs for epilepsy, including felbamate (Felbatol), topirimate (Topamax), lacosamide (Vimpat), and retigabine (Potiga)^17^. In the ADD program, maximal electroshock seizure (MES) and chemical induced seizures (scPTZ) along with toxicity screen (rotorod in mice, positional sense and gait in rats) are employed to screen the new anticonvulsants, which gradually became the most widely used animal models world-wide for the discovery of new anticonvulsant drugs at the initial stage^18–20^. Except the *in vivo* models, some mechanistically related *in vitro* methods are also used to evaluate the anticonvulsant potential via obtaining the compound’s affinity with excitatory (glutamate), inhibitory (GABA) receptor and other related enzymes^21^^,^^22^.

**Figure 1.**
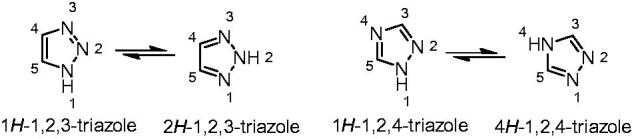
The structures of triazole.

A triazole refers to the heterocyclic compounds with molecular formula C_2_H_3_N_3_, having a five-membered ring of two carbon atoms and three nitrogen atoms. There are two sets of isomers that differ in the relative positions of the three nitrogen atoms. Each of these has two tautomers that differ by which nitrogen has a hydrogen bonded to it (Figure 1). The aromaticity and electron rich property of triazole endows it to readily bind with various kinds of enzymes and receptors via weak interactions such as hydrogen bonds, coordination bonds, ion-dipole, cation-π, hydrophobic effect, van der Waals force and so on, and thus makes it widely employed in various field^23^^,^^24^.

The triazole ring is also an important isostere of oxazole, thiazole, imidazole, pyrazole, and so on. Based on the excellent properties of triazole, a huge number of triazole-based derivatives have been prepared and studied for their biological activities, especially for the research and development of new drugs^25–30^.

Since the approval and sales of triazolam and alprazolam (which have triazole moiety) as anticonvulsants in the 1980s, more and more medicinal chemists paid their attention to design and synthesis the triazole-derivatives for their anticonvulsant activity. Recently, several scholars have reviewed the medicinal attributes of triazoles^23^^,^^28^^,^^31–34^, part of which focused on the anticonvulsant agents^23^^,^^34^. However, these reviews focus on the work of part laboratories and short time periods, which limited the overall and systematic understanding of the medicinal attributes of triazoles in anticonvulsant agents for readers. In view of this, we intended to review the triazole derivatives with anticonvulsant activity systematically and comprehensively from the reported literatures in recent 20 years. These researches can be classified into the following categories: (1) 1-substituted-1,2,4-triazoles, (2) 4-disubstituted-4*H*-1,2,4-triazoles, (3) polysubstituted-4*H*-1,2,4-triazoles, (4) substituted-triazolones and substituted-triazolthiones, (5) fused-triazoles, (6) fused-triazolones, and (7) 1,2,3-triazoles, according to the difference of structures.

## 1. 1-Substituted-1,2,4-triazoles

**Scheme 1.**
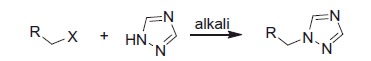
The introducing method of triazole for 1-substituted-1,2,4-triazoles.

**Figure 2.**
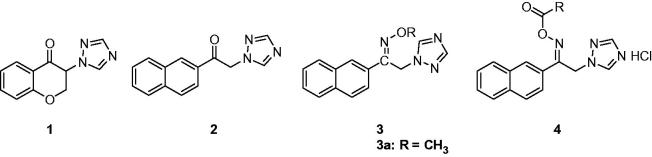
Structures of 1-substituted-1,2,4-triazoles (**1**–**4**).

1-Substituted-1,2,4-triazoles, usually prepared by the reaction of halide and 1,2,4-triazoles (Scheme 1), were reported frequently as anticonvulsive compounds. In 2006, Shafiee’s team reported several triazolylchromans as novel anticonvulsant agents. The anticonvulsant activities of these compounds were evaluated by determining seizure latency and protective effect against pentylenetetrazole (PTZ)-induced seizures in mice. Among of them, 3-(1*H*-1,2,4-triazol-1-yl)chroman-4-one (**1**, Figure 2) exhibited significant activity in delaying seizures as well as effective protection against PTZ-induced hind limb tonic extension (HLTE), and deaths at a dose of 5 mg/kg^35^. To further confirm the treatment for epilepsy of triazolylchromans, the lithium-epilocarpine induced seizure and PTZ-induced kindling models were used to study the anticonvulsive and antiepileptogenic properties of compound **1**. The results suggested that compound **1** showed highly effective at the dose of 5 mg/kg against acute PTZ-induced convulsions, while exhibited limited effects in PTZ-induced kindling model which had considerable effect on seizure index only at the highest dose of 30 mg/kg. The effectiveness of compound **1** against lithium-epilocarpine induced status epilepticus, suggesting the potential application of it in the treatment of status epilepticus^36^.

In 1988, Dalkara et al. synthesised 1-(2-naphthyl)-2-(1,2,4-triazol-1-yl)ethanone (**2**, Figure 2), which has a triazole instead of an imidazole ring in the nafimidone (an anticonvulsant possessing activity profile similar to phenytoin or carbamazepine). It possessed noticeable anticonvulsant activity at dose of 30 mg/kg in the PTZ model^37^. As a continue of the above work, they prepared some new oxime ether derivatives (**3**, Figure 2) of 1-(2-naphthyl)-2-(1,2,4-triazol-1-yl)ethanones and tested their anti-MES and anti-PTZ activities. The pharmacological experiment suggested that the size and structure of the O-substituents in oxime ethers were found to be important for the activity. Compounds with small alkyl groups such as methyl, ethyl, propyl, allyl were active in 100 mg/kg dose levels, whereas larger groups such as benzyl and substituted benzyl molecules resulted in a lack of anticonvulsant activity and a decrease in toxicity. Compound **3a** (with R = CH_3_) was the most active one, which exhibited significant protection activity against MES and PTZ induced seizure in 0.5 and 4 h interval, at the dose of 100 mg/kg^38^.

Based on the potent anticonvulsant activity of the oxime ester derivatives of nafimidone containing triazole, Sari et al. prepared 14 novel 1-(2-naphthyl)-2-(1*H*-1,2,4-triazol-1-yl)ethanone oxime ester derivatives (**4**, Figure 2) through modifications on the oxime ester function. Their anticonvulsant and neurotoxic effects were evaluated in mice using MES, PTZ and rotarod tests. In order to get insights into the effects of these compounds on VGSCs and Atype GABA receptors (GABAARs), a docking studies using homology model of Na^+^ channel inner pore and GABA_A_R were carried out in this study. It was found that the compounds bound VGSCs in similar ways as phenytoin, carbamazepine, and lamotrigine, and showed strong affinity to benzodiazepine (BZD) binding site. Pharmacology test showed that some of these compounds displayed anti-MES and anti-PTZ activity at 30 or 100 mg/kg^39^.

**Figure 3.**
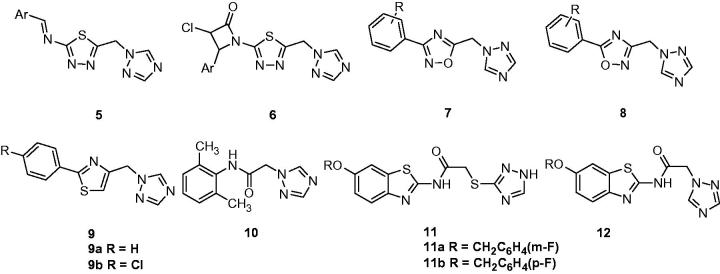
Structures of 1-substituted-1,2,4-triazoles (**5**–**12**).

Srivastava et al. described the synthesis and anticonvulsant activity of several 1-disubstituted-4*H*-1,2,4-triazoles bearing thiadiazole moiety (**5 and 6**, Figure 3). All compounds showed anticonvulsant activities against PTZ induced seizures with 20–80% protection^40^.

A series of 3- and 5-aryl-1,2,4-oxadiazoles containing triazole (**7** and **8**, Figure 3) were prepared and tested for anticonvulsant activity in a variety of models. These compounds showed a remarkable *in vivo* activity against electrically and chemically induced seizures. Compound **8a** (R = *p* − *F*) gave a median effective dose (ED_50_) of 15.2 mg/kg in the rat PTZ test in *p.o*. Additionally, a sufficient therapeutic index (PI) was achieved by **8b** (R = H) with a PI value bigger than 13 and 24 for PTZ and MES models, respectively. Mechanism studies on selected compounds revealed that the modulating of the GABA receptor was via a binding site but not the benzodiazepine site, and sodium channel blocking are both involved in the mechanisms^41^.

As a part of continuous investigation into the area of (arylalkyl)azole anticonvulsants, several new thiazole incorporated (arylalkyl)azoles were designed and prepared. Target compounds were screened for their anticonvulsant properties using MES and PTZ models in mice. Among them, 1-[(2-phenylthiazol-4-yl)methyl]-1*H*-1,2,4-triazole (**9a**, Figure 3), and its 4-chlorophenyl analog (**9b**, Figure 3) were able to display noticeable anticonvulsant activity in both PTZ and MES tests at the dose of 30 and 100 mg/kg^42^.

A group of N-phenylacetamide and N-phenylpropanamide derivatives bearing 1,2,4-triazole at ω-position were synthesised and their anticonvulsant activity was evaluated in the MES test. The most active compound was 2-(1*H*-1,2,4-triazole-1-yl)-N-(2,6-dimethylphenyl)acetamide (**10**, Figure 3), which exhibited 87.5% protection against electroshock induced seizures at 100 mg/kg dose in mice at 0.5 and 4 h^43^.

Recently, new benztriazoles with a mercapto-triazole/triazole were synthesised and evaluated for their anticonvulsant activity and neurotoxicity by using the maximal electroshock (MES), subcutaneous pentylenetetrazole (scPTZ), and rotarod neurotoxicity (TOX) tests. The results demonstrated that the compounds possessing 1,2,4-triazole-3-thiol (**11**, Figure 3) displayed the better anticonvulsant activity than that of compounds possessing 1,2,4-triazole (**12**, Figure 3). Compounds **11a** and **11b** (Figure 3) showed an ED_50_ value of 50.8 and 54.8 mg/kg in the MES test and 76.0 and 52.8 mg/kg in the scPTZ seizures test, respectively^44^.

**Figure 4.**
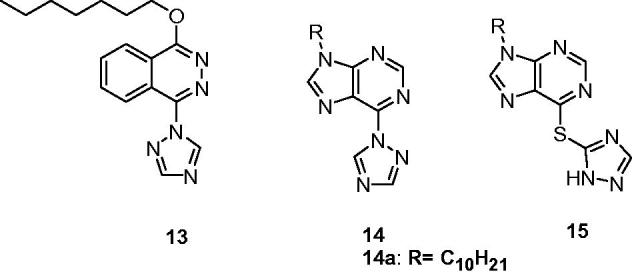
Structures of 1-substituted-1,2,4-triazoles (**13**–**15**).

Except the N-(arylalkyl)triazoles’ anticonvulsant activities, some N-aryltriazoles were also reported for their anticonvulsant activities. Wang and co-workers screened a series of 1-alkoxy-4-(1*H*-1,2,4-triazol-1-yl)phthalazine derivatives against MES induced seizures in mice. Among the tested compounds, **13** (Figure 4) was the most active compound with an ED_50_ of 28.9 mg/kg, median toxicity dose (TD_50_) of 173.6 mg/kg, and PI of 6.0^45^.

Several 9-subsituted-6-(1*H*-1,2,4-triazol-1-yl) (**14**, Figure 4) and (3*H*-1,2,4-triazol-3-ylthio)-9*H*-purines (**15**, Figure 4) were synthesised as new anticonvulsant agents. The results of MES tests indicated that the series of **14** showed good activity, while the series of **15** did not show protection at the dose of 100 mg/kg. Compounds **14a** was the most potent compound, with an ED_50_ of 23.4 mg/kg and a PI value of more than 25.6 after intraperitoneal administration in mice. Furthermore, it showed significant oral activity in MES test in mice, with an ED_50_ of 39.4 mg/kg and a PI above 31.6^46^.

## 2. 4-Disubstituted-4*H*-1,2,4-triazoles

**Scheme 2.**
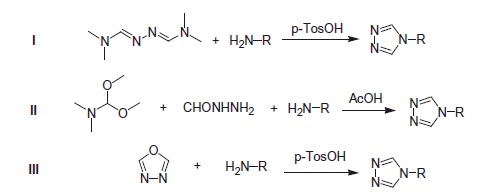
The introducing method of triazole for 4-Substituted-1,2,4-triazoles.

4-Disubstituted-1,2,4-triazoles usually are prepared by three method (as seen in Scheme 2): one is via the reaction of N, N-dimethylformamide azine with primary amines mediated by *p*-toluene sulfonic acid to give the 4-disubstituted-1,2,4-triazoles^47^; the other one is developed by Michael, utilising a wide range of substituted primary amines (arylamine or aliphatic amine), acyl hydrazines, and dimethylformamide dimethyl acetal^48^; the last one is through the reaction of 1,3,4-oxadiazole with the primary amines^49^. 4-Disubstituted-1,2,4-triazoles can also be categorised into 4-aryl-triazoles and 4-alkyl-triazoles. Both were reported the potent anticonvulsant activity.

**Figure 5.**
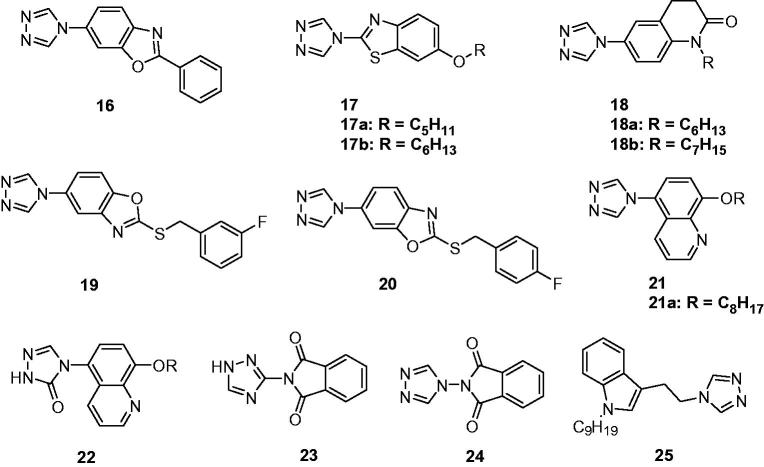
Structures of 4-substituted-1,2,4-triazoles (**16**–**25**).

Quan’s team reported the design and synthesis of numbers of triazolebenzo[d]oxazoles as anticonvulsant agents. Among of them, 2-phenyl-6-(4*H*-1,2,4-triazol-4-yl)benzo[d]oxazole (**16**, Figure 5) was the most active and also had the lowest toxicity. In the anti-MES potency test, it showed median effective dose (ED_50_) of 29.5 mg/kg, a median toxicity dose (TD_50_) of 285 mg/kg, and a protective index (PI) of 9.7, which is greater than the reference drug, carbamazepine, whose PI value was 6.4. This attempt suggested that the mono substituted triazoles also meet the request of the anticonvulsant activity^50^.

Based on the expectation that combining the anticonvulsant activity of benzothiazole and triazole, a series of triazole derivatives (**17**, Figure 5) containing benzothiazole were synthesised by Quan’s team. The compounds tested in the MES screens were all effective. Among of which, the most promising compound was identified as **17a** (Figure 5), with an ED_50_ value of 39.4 mg/kg and a PI value of 17.3. It is worth mentioning that these compounds had long duration of anticonvulsant activity with the **17b** (Figure 5) keeping protection activity in MES test more than 6 h. Moreover, compound **17a** significantly inhibited the clonic seizures, tonic seizures and lethality with the rates of 80, 100, and 100%, respectively; and the result of PTZ test indicated that compound **17a** could be used as a lead compound for the development of drugs treating absence seizures^51^.

Deng et al. embarked on a program to combine the antidepressant and anticonvulsant activities of 3,4-dihydro-2(1*H*)-quinolinone and triazole through the preparation of some triazole-containing quinolinones (**18**, Figure 5). The pharmacology screen results showed that several compounds showed antidepressant and anticonvulsant activities. Compounds **18a** and **18b** (Figure 5) showed 100% protection against MES-induced seizures at the dose of 100 mg/kg. No neurotoxicity was found at the dose of 100 mg/kg^52^.

Recently, a series of benzo[d]oxazoles containing triazole were designed and synthesised as new anticonvulsant agents. The pharmacology results showed that most compounds exhibited anticonvulsant activity in MES and Sc-PTZ models. Among them, compound **19** (Figure 5) was the most potent with ED_50_ value of 11.4 and 31.7 mg/kg in MES and Sc-PTZ models, respectively. The TD_50_ value of **19** was 611.0 mg/kg, which resulted in the protective index (PI) value of 53.6 and 19.3. Further, the pre-treatment of thiosemicarbazide (an inhibitor of γ-aminobutyric acid synthesis enzyme) significantly decreased the activity of **19** in MES, which suggested that the GABAergic system may contribute at least in part to the anticonvulsive action^53^. Meanwhile, another series of triazole-containing benzo[d]oxazoles were prepared *via* altering the position of triazole. In this study, compound **20** was obtained with an ED_50_ of 12.7 mg/kg and 29.5 mg/kg in MES and Sc-PTZ models, respectively. The rotarod test showed the TD_50_ of 491.0 mg/kg for **20**^54^.

Two series of 8-alkoxy-5-(4*H*-1,2,4-triazol-4-yl)quinolines (**21**, Figure 5) and 8-alkoxy-5-(2*H*-1,2,4-triazol-3-one-4-yl)quinolines (**22**, Figure 5) were synthesised by Wang and his co-workers. Among the synthesised compounds, **21a** (Figure 5) was the most active compound with ED_50_ of 8.80 mg/kg, TD_50_ of 176.03 mg/kg, and PI of 20.0. Its neurotoxicity was lower than all other synthesised compounds as well as that of the reference drug carbamazepine. The replacement of triazole by triazolone markedly decreased the activity with only two active at the large dose of 300 mg/kg^55^.

Several 2-arylisoindoline-1,3-dione derivatives were prepared and evaluated for their anticonvulsant activities. The *in vivo* screening data indicated that the 4-triazoly derivative (**23**, Figure 5) was the most promising one, which could increase the tonic seizure threshold significantly in the PTZ model. Docking studies using the model of sodium channel has revealed that compound **23** interacted mainly with residues II-S6 of NaV1.2 by making hydrogen bonds and had additional hydrophobic interactions with domain I and II in the channel’s inner pore^56^.

Another series of 2-arylisoindoline-1,3-dione derivatives were prepared by reacting of phthalic anhydride and various amines. Among of them, 2-(3*H*-1,2,4-Triazole-3-y) isoindoline-1,3-dione (**24**, Figure 5) administered intraperitoneally (*i.p.*) at a dose of 0.2 ml/kg, were found significantly delayed the onset and antagonised picrotoxin-induced seizures^57^.

Recently, Quan et al. designed and synthesised several novel triazoleindole derivatives with tryptamine as the starting material. Among the compounds studied, compound **25** (Figure 5) was found to be the most potent compound with an ED_50_ value of 10.2 mg/kg in MES test in intraperitoneal administrated mice, possessed better anticonvulsant activity and higher safety than carbamazepine^58^.

## 3. Polysubstituted-4*H*-1,2,4-triazoles

Some polysubstituted-1,2,4-triazoles (include the bis-substituted) were prepared and screened for their anticonvulsant activities. These studies enriched the structure-anticonvulsant activity relationship of triazoles and indeed provided a large amount of compounds with prominent anticonvulsant effects.

**Figure 6.**
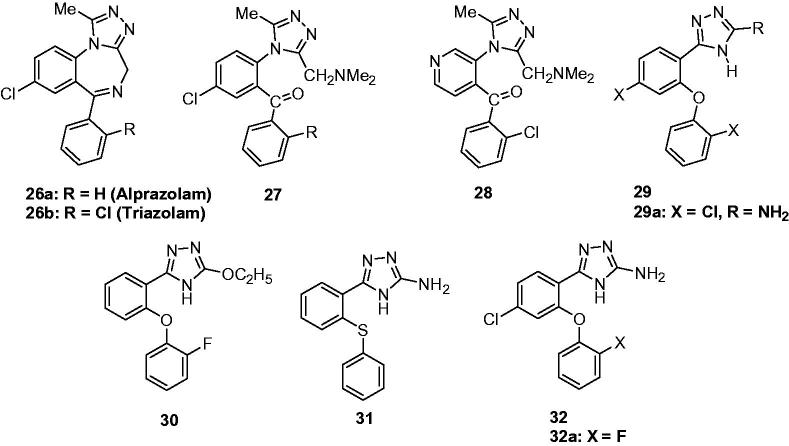
Ring-opening derivatives of alprazolam and triazolam (**26**–**32**).

Alprazolam and triazolam were two of the promising anticonvulsant agents that containing triazole (**26a** and **26b**, Figure 6). Around the triazole-benzodiazepine structure, many analogues were prepared to find new anticonvulsant agents. Gall et al. reported a type of 5-chloro-2-[3-[(dimethylamino)methyl]-5-methyl-4*H*-1,2,4-triazol-4-yl]benzophenones (**27**, Figure 6), which exhibited potent sedative, muscle relaxant and anticonvulsant activities^59^. As a isosteric molecule of compound **27**, 3-[3-[(dimethylamino)methyl-5-methyl-4*H*-1,2,4-triazol-4-yl]-4-(o-chlorobenzoyl)-pyridine (**28**, Figure 6) was prepared by Knaus’s team. Based on the PTZ models, compound **28** exhibited good antivonvulsant activity with an ED_50_ of 7.84 mg/kg^60^.

Amongst all pharmacodynamic models suggested for binding to the benzodiazepine receptor at least two features are common: an aromatic ring and a coplanar proton accepting group in suitable distance. Also, the presence of a second out-of-plane, aromatic ring could potentiate binding to the receptor. Abbas Shafiee and his team launched a project to design simple non-rigid structures with benzodiazepine activity based on the proposed SAR. A series of 4H-3-(2-phenoxy)phenyl-1,2,4-triazole derivatives (**29**, Figure 6), with a simple non-rigid structure, were designed, which had all the suggested requirements for binding to the benzodiazepine receptors. Their benzodiazepine effects and anticonvulsant activity were evaluated. Among the tested compounds, compound **29a** with dichloro-substituent showed the best anticonvulsant activity with an ED_50_ of 12.0 mg/kg in the PTZ models. To clarify whether the designed compounds could mimic the structure of a benzodiazepine agonist, conformational analysis on designed molecules as well as a known benzodiazepine agonist estazolam was performed followed by superimposition of energy minima conformers. The results showed that the main proposed pharmacofores were well matched. In addition, the activity of the compounds is significantly reduced by flumazenil, a benzodiazepine antagonist, which further confirms that this effect is mediated through benzodiazepine receptors.

From the pharmacological results, the follow SARs were obtained: Analogous with chloro substituent on position 2 of phenoxy group and position 4 of phenyl ring are more potent than the corresponding unsubstituted compounds. These two positions are well matched to positions 2′ and 7 of the benzodiazepine ring; it has been established that electron withdrawing substituent on these positions enhance the activity.

The study indicates that some synthesised 1,2,4-triazoles with a simple non-rigid structure can also show benzodiazepine activity comparable with diazepam, which lead us to the new class of benzodiazepine receptor ligands^61^.

Followed the above study, several new 2-substituted-5-[2–(2-fluorophenoxy)phenyl]-1,3,4-oxadiazoles and 1,2,4-triazoles were prepared to explore the necessity or not of the 2-NH_2_ for the anticonvulsant activity. The results suggested that compounds with substituents other than amino group did not have significant activity in PTZ and MES models. Only compounds with OEt (**30**, Figure 6) showed mild effects in PTZ test with an ED_50_ of 84.84 mg/kg^62^.

Bioisosterism is a good method to obtain derivatives with similar or better activity. With the purpose to evaluate the effects of different substitutents on pharmacological activity, a series of 1,2,4-triazole derivatives were prepared by bioisosteric replacement of oxygen with sulphur in the compounds **30**. Unfortunately, none of them, except (**31**, Figure 6) showed anticonvulsant effect in dose of 100 mg/kg^63^.

Two novel 3-amino-5-(4-choloro-2-phenoxyphenyl)-4*H*-1,2,4-triazole derivatives (**32**, Figure 6) were prepared as the derivatives of compound **31** based on the previous SARs. In the PTZ model, compound **32a** (Figure 6) showed potent anticonvulsant activity (ED_50_ = 1.4 mg/kg) compared to diazepam (ED_50_ = 1.2 mg/kg)^64^.

**Figure 7.**
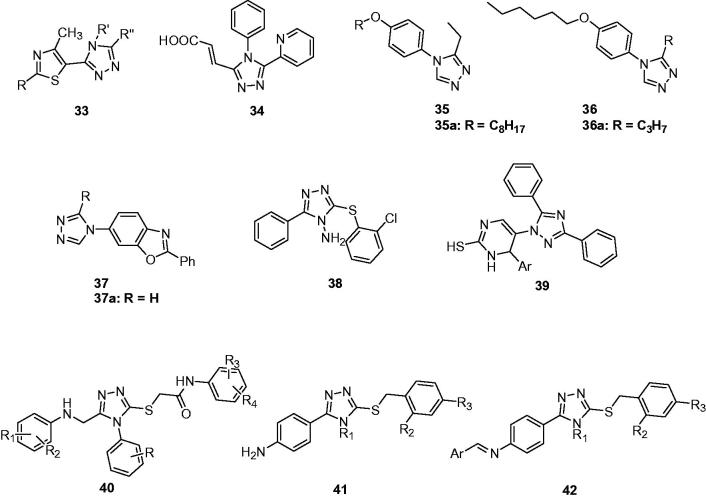
Polysubstituted-4*H*-1,2,4-triazoles (**33**–**42**).

Several new N-4-substituted triazolylthiazoles (**33**, Figure 7) were prepared and screened for anticonvulsant activity against PTZ-induced seizures. Most compounds exhibited protection against PTZ-induced seizures in the range doses of 25–200 mg/kg. Pre-treatment of animals with flumazenil (10 mg/kg, *i.p.*) as a benzodiazepine receptors antagonist did not have any significant effect on their anticonvulsant activity, which suggested that the anticonvulsant activity of N 4-substituted triazolyl thiazoles is not mediated by activation of benzodiazepine receptors^65^.

In another work, a groups of 3-(3,4-diaryl-1,2,4-triazole-5-yl)propenoic acid derivatives were synthesised by condensation of N3-substituted amidrazones with *cis*-butenedioic anhydride. Compound **34** (Figure 7) was subjected to the PTZ-induced seizures and exhibited protection in the dose of 50 and 100 mg/kg^66^.

Quan’ team designed and prepared a series of 4-(4-alkoxylphenyl)-3-ethyl-4*H*-1,2,4-triazole derivatives (**35**, Figure 7) as ring-opening analogue of 7-alkoxy-4,5-dihydro[1,2,4]triazolo[4,3-a]quinolines. They expected that a rotatable triazole ring may have higher affinity for the receptor and enhance their anticonvulsant activity. Compound **35a** (Figure 7) was chose as the most promising one, which showed strong anticonvulsant activity and the best PI value in MES test, with an ED_50_ of 8.8 mg/kg and a PI value of 9.3. What’s more, compound **35a** was effective against the seizures induced by PTZ, isoniazid, 3-mercaptopropionic acid and thiosemicarbazide with ED_50_ values of 26.4, 38.1, 31.7, and 22.8 mg/kg, respectively^67^.

In order to discuss the effect of different substituted groups in the third position of 4-(4-alkoxyphenyl)-4*H*-1,2,4-triazoles to anticonvulsant activity, a series of 3-substituted-4-(4-hexyloxyphenyl)-4*H*-1,2,4-triazoles (**36**, Figure 7) was designed and prepared. Their anticonvulsant activities and neurotoxicity were evaluated by MES test and rotarod assay, respectively. Compound **36a** with *n*-propyl was the most active compound amongst all 19 derivatives, with an ED_50_ value of 5.7 mg/kg, which was greater than that of the reference drug phenytoin. In addition, it was also safer than phenytoin with a PI value of 11.5^68^.

In another work, a series of 6-(3-substituted-4*H*-1,2,4-triazol-4-yl)-2-phenylbenzo[d]oxazoles (**37**, Figure 7) was synthesised by Quan’s team. The anticonvulsant effect and neurotoxicity of the compounds were evaluated with MES test, PTZ, and rotarod tests in intraperitoneally injected mice. Among the synthesised compounds, compound **37a** could be considered the potentially most useful and safe therapeutic compound, with ED_50_ =  29.6 mg/kg, TD_50_ = 285 mg/kg, and PI = 9.7^69^.

Sahoo et al. reported a series of 3,5-substituted-2-amino-1,2,4-triazole derivatives as anticonvulsant agents. All the compounds were screened for their anticonvulsant activity in the MES model and were compared with phenytoin sodium and carbamazepine. Some of the tested compounds showed comparable anti-MES activity to phenytoin and carbamazepine after 0.5 h. And compound **38** was more potent than carbamazepine after 4 h^70^.

A group of 1,3,5-substituted-1*H*-1,2,4-triazoles (**39**, Figure 7) were prepared by Khanage et al. Their anticonvulsant activity was evaluated by the MES model. Four of them showed comparable anti-MES activity to phenytoin and carbamazepine with protection at the dose of 30 mg/kg after 0.5 h^71^.

A series of 4-phenyl-5-phenylaminomethyl-1,2,4-triazol-(4*H*)-3-ylmercaptoacetic acid anilides (**40**, Figure 7) were designed and prepared including several pharmacophores. In PTZ model with mice, compounds had moderate anticonvulsive activity, but less than that of lamotrigine. The SAR analysis suggested the halogen substitution is necessary for the anticonvulsant of these compounds^72^.

In another study, some 3-(arylalkylthio)-4-alkyl/aryl-5–(4-aminophenyl)-4*H*-1,2,4-triazole derivatives (**41** and **42**, Figure 7) were synthesised and screened for their anticonvulsant activity. Some valuable compounds have emerged with activity in the dose of 100 mg/kg in both MES and scMET tests at 0.5 and 4 h. The obtained results demonstrated that either small alkyl substitution at triazole ring or primary aromatic amino group were essential for bioactivity. The replacement of small alkyl by aryl substitution at the same position completely blocked activity in both MES and scPTZ models. The complete loss of activity was observed when the NH_2_ function was replaced with an arylideneamino (–N=CH–Ar) moiety^73^.

## 4. Substituted-triazolones and substituted-triazolthiones

**Scheme 3.**
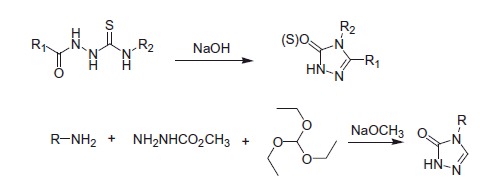
The prepared methods of triazolones and triazolthiones.

Classical methods for the synthesis of triazolones and triazolthiones are by heating the substituted isothiosiyanates or semicarbazide in the presence of alkaline medium (Scheme 3). Another method for preparing triazolones is utilising a wide range of substituted primary amines (arylamine or aliphatic amine), methyl carbazate, and triethyl orthoformate in the presence of alkaline medium (Scheme 3). Many triazolones and triazolthiones were prepared by the two methods and reported with the potent anticonvulsant activity.

**Figure 8.**
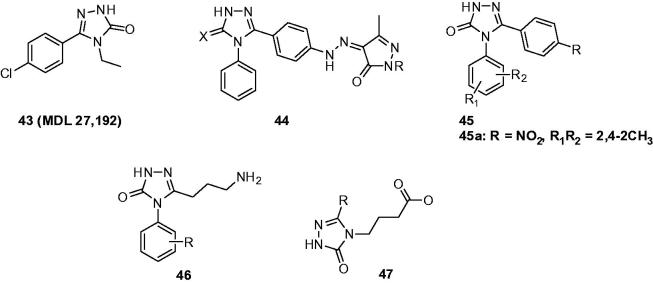
Substituted triazolones (**43**–**47**).

In 1997, Kehne et al. carried out a research aiming to provide functional evidence for MDL 27,192’s profile (**43**, Figure 8) as a potential broad spectrum anticonvulsant with neuroprotective activity. In this work, MDL 27,192 displayed potential anticonvulsant activity in a wide range of epilepsy models included audiogenic seizures in the seizure susceptible DBA/2J (ED_50_ =  14.6 mg/kg) or Frings mouse (ED_50_ =  9.6 mg/kg), spike wave seizures in genetic absence epilepsy rats of Strasbourg (effective in 60 mg/kg), MES seizures in mice (ED_50_ =  45.3 mg/kg) and rats (ED_50_ =  8.2 mg/kg), corneally kindled seizures in rats (ED_50_ =  24.5 mg/kg) and chemically induced seizures in CD-1 mouse (bicuculline (ED_50_ =  27.9 mg/kg), PTZ (ED_50_ =  12.2 mg/kg), picrotoxin (ED_50_ =  51.5 mg/kg), 3-mercaptopropionic acid (ED_50_ =  45.3 mg/kg), quinolinic acid (ED_50_ =  15.9 mg/kg), and strychnine (ED_50_ =  51.5 mg/kg). When compared to valproate, orally administered MDL 27,192 was 17- to 48-fold more potent as an anticonvulsant and showed a safety index one to 3-fold greater^74^.

A series of 5-pyrazolinones containing triazolone or triazolthione (**44**, Figure 8) were prepared by Küçükgüzel and his coworkers. These compounds were tested at 100 mg/kg in pentylenetetrazole induced seizures in mice, and protections ranging from 10% to 40% were obtained^75^.

Yogeeswari’ team always devotes to the research of various aryl-substituted semicarbazones as potential anticonvulsant agents. Based on the heat of triazole moiety in the study of anticonvulsants, they launched a program to cyclise these aryl semicarbazones, which would lead to 1,2,4-triazoles. A new series of 4,5-diphenyl-2*H*-1,2,4-triazol-3(4*H*)-one (**45**, Figure 8) were synthesised, and four animal seizures models [maximal electroshock seizure (MES), subcutaneous pentylenetetrazole (scPTZ), subcutaneous strychnine (scSTY), and subcutaneous picrotoxin (scPIC)] were conducted to evaluate their anticonvulsant activities. The effect of cyclisation of semicarbazones template depended on the substituents. Some more potent derivatives with 2,4/2,5-dimethylphenyl groups were obtained, but the activity was decreased or maintained for the 2,6-dimethyl/4-fluorophenyl derivatives. compound **45a** (Figure 8) had increased the GABA level more than 10 times compared to the control in rat brain at 100 mg/kg, which indicated that the cyclised aryl semicarbazones (4,5-diphenyl-2*H*-1,2,4-triazol-3(4*H*)-ones) exhibited anticonvulsant activity via GABA-mediation^76^.

In the next work, Yogeeswari and coworkers prepared several 1,2,4-triazolone derivatives integrated with GABA with the purpose of investigating their antiepileptic activities. All the compounds were evaluated against MES and PTZ induced seizures at dose of 100 and 300 mg/kg intraperitoneally in mice. Interestingly, 4-aryl substituted 1,2,4-triazolones (**46**, Figure 8) showed activity in both models while 5-aryl substituted 1,2,4-triazolones (47, Figure 8) were devoid of any anticonvulsant activities^77^.

**Figure 9.**
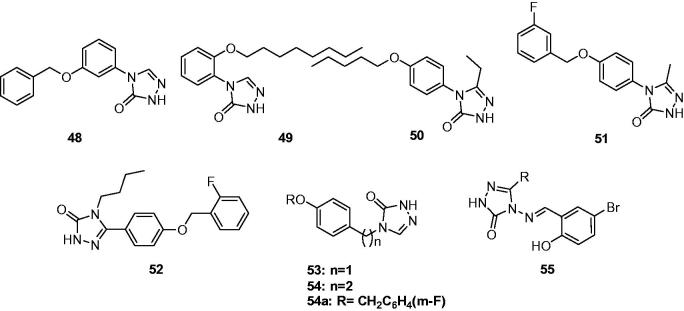
Substituted triazolones (**48**–**55**).

A series of alkoxyaryltriazolones were synthesised by Quan’s team, and tested for their anticonvulsant activity and neurotoxicity in mice by MES test and rotarod test, respectively. All target compounds exhibited anticonvulsant activity in varying degrees in the MES test. 4-(3-Benzyloxy-phenyl)-2,4-dihydro-[1,2,4]triazol-3-one (**48**, Figure 9) was the most promising compound with an ED_50_ value of 30.5 mg/kg and a TD_50_ value of 568.1 mg/kg, which gave a protective index value (PI) of 18.63. Quan et al. suggested that these alkoxytriazolones acted via inhibiting of voltage-gated ion channels and modulating of GABAergic activity^78^.

In another work, several new alkoxyaryltriazolones were prepared as anticonvulsant agents by altering the site of alkoxy group of **48**. Based on the results, 4-(2-octyloxy-phenyl)-2,4-dihydro-3*H*-1,2,4-triazol-3-one (**49**, Figure 9) was the most promising compound with the ED_50_ of 23.7 mg/kg, the TD_50_ of 611.0 mg/kg, and the protective index (PI) of 25.8. In addition, the potency of compound **49** against seizures induced by pentylenetetrazole, 3-mercaptopropionic acid, and bicuculline were also established in this work^79^.

To obtain more structure–activity relationship of alkoxyaryltriazolones, the group of Quan prepared some 4-(4-alkoxyphenyl)-3-ethyl-1*H*-1,2,4-triazol-5(4*H*)-one derivatives and evaluated their anticonvulsant activity by MES model. In which, 3-ethyl-4-(4-(pentyloxy)phenyl)-1*H*-1,2,4-triazol-5(4*H*)-one (**50**, Figure 9) was the most potent compound with the ED_50_ value of 26.9 mg/kg, and protective index (PI) value of 11.0^80^.

Meanwhile, another series of 4-(4-alkoxyphenyl)-1*H*-1,2,4-triazol-5(4*H*)-ones were prepared and evaluated the anticonvulsant activity in MES test in mice. Among of which, compound **51** (Figure 9) was found to possess better anti-MES activity and higher safety than marketed drug carbamazepine with an ED_50_ value of 25.5 mg/kg and protective index (PI) value above 48.8^81^.

A series of 4-butyl-5-(4-alkoxyphenyl)-2*H*-1,2,4-triazol-3(4*H*)-ones were also synthesised and screened for their anticonvulsant effects by MES test. Among the synthesised compounds, compound **52** (Figure 9) was the most potent with ED_50_ value of 27.4 mg/kg and a protective index value of 12.0. Besides the anti-MES efficacy, the potency of **52** against seizures induced by pentylenetetrazole, 3-mercaptopropionic acid, and bicuculline was also established, which suggested that enhancing of GABAergic activity might be involved in its mechanisms of action^82^.

Recently, several 4-(4-methoxybenzyl/phenethyl)-2*H*-1,2,4-triazol-3(4*H*)-ones derivatives (**53** and **54**, Figure 9) were prepared to find new anticonvulsant agents among triazolones. The results of MES test revealed that compounds **54** exhibited superior anti-MES activity than compounds **53**. Among the compounds synthesised, compound **54a** with R = CH_2_C_6_H_4_ (*m*-F) was the most potent compound, with a median effective dose of 19.5 mg/kg and a protective index of 5.1 after intraperitoneal administration in mice^83^.

A series of 1,2,4-triazole-3-one derivatives (**55**, Figure 9) bearing the salicyl moiety were synthesised under microwave irradiation conditions, and their anticonvulsant activities were evaluated by the Anticonvulsant Screening Program of the National Institute of Health, USA. Some of the compounds had moderate anticonvulsant activities in MES model and minimal clonic seizure model (6 Hz model) in mice (protection against MES induced seizures at 30 and 100 mg/kg). No neurotoxic effect was found at the dose of 100 mg/kg^84^.

**Figure 10.**
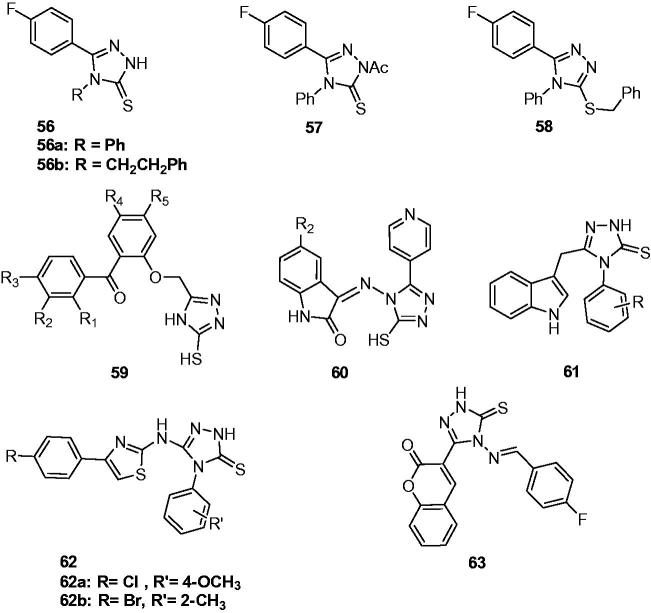
Substituted triazolthiones (**56**–**63**).

Guelerman et al. synthesised a series of 5-(4-fluorophenyl)-4-substituted-2,4-dihydro-3*H*-1,2,4-triazole-3-thiones (**56**, Figure 10) and their N-acetyl (**57**, Figure 10) and S-benzyl derivatives (**58**, Figure 10). Their anticonvulsant activity was determined against PTZ-induced seizures. Among them, compounds **56a**, **56b**, **57**, and **58** exhibit anticonvulsant activity^85^.

Khanum reported the synthesis of 3–(2-aroylaryloxy)methyl-5-mercapto-4*H*-1,2,4-triazole analogues (**59**, Figure 10) by intramolecular cyclisation of (2-aroylaryloxy)acetates with thiosemicarbazide. The anticonvulsant activities of these compounds were screened by MES model in rats. The results showed that compounds with chloro in the R_2_ and R_4_ substituents were more active than others and showed comparative activity to that of phenytoin^86^.

A series of 5-substituted Isatin-imino derivatives (**60**, Figure 10) containing triazolthione was prepared for their anticonvulsant properties by Pandeya et al. Some of them showed the anti-MES and anti-PTZ activities^87^.

A series of new 5-(1*H*-indol-3-yl)methyl-4-(substituted aryl)-2,4-dihydro-3*H*-1,2,4-triazole-3-thiones (**61**, Figure 10) were prepared to screen their anticonvulsant and toxicity. Some compounds showed comparable MES activity to phenytoin and carbamazepine after 0.5 h^88^.

Thiazole and triazole moieties were clubbed together to get several 3-[4-(substituted phenyl)-1,3-thiazol-2-ylamino]-4-(substituted phenyl)-4,5-dihydro-1*H*-1,2,4-triazole-5-thiones (**62**, Figure 10). Their anticonvulsant activity *in vivo* was screened by MES and scPTZ models. Two compounds **62a** and **62b** (Figure 10) showed significant anticonvulsant activity in both the screens with ED_50_ values 23.9 and 13.4 mg/kg, respectively, in MES screen and 178.6 and 81.6 mg/kg, respectively, in scPTZ test. They displayed a wide margin of safety with PI value of 18.3 and 51, respectively, which were much higher than the standard drugs^89^.

Bhat et al. reported the synthesis and anticonvulsant properties of coumarin incorporated 1,2,4-triazole-5-thione derivatives. Compound **63** (Figure 10) showed significant anticonvulsant activity. In MES test, compound **63** showed protection from seizures at the dose of 30 mg/kg after 0.5 h and it kept the activity after 4.0 h at higher dose of 100 mg/kg. In the scPTZ screen, compound **63** was active at 100 mg/kg after 0.5 h, and was also found to be active after 4.0 h at higher dose 300 mg/kg^90^.

**Figure 11.**
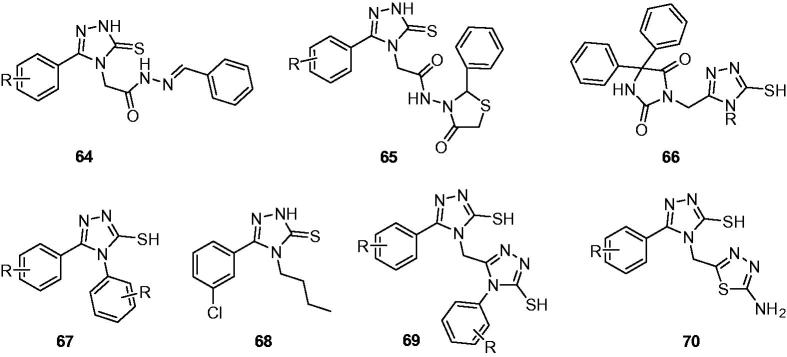
Substituted triazolthiones (**64**–**70**).

Kumudha et al. synthesised numbers of acetohydrazide Schiff’s bases containing 1,2,4-triazole-5-thione (**64**, Figure 11), which was further cyclised with thioglycolic acid to get some thiazolidinones (**65**, Figure 11). Parts of these compounds were evaluated for anticonvulsant, CNS depressant activities and neurotoxicity. All the tested compounds showed potent anticonvulsant activity, with good protection against PTZ and MES induced seizures at 20 mg/kg. No toxicity was found at the same dose in the rotarod test^91^.

Some new phenytoin-triazole derivatives (**66**, Figure 11) were prepared using hybrid approach as anticonvulsant agents. These compounds exhibited weaker anticonvulsant activity that phenytoin in the PTZ model, but comparative protection in the MES test^92^.

A series of substituted 4,5-diphenyl *4H*-1,2,4-triazole-3-thiols (**67**, Figure 11) have been synthesised by Kumudha et al. Some of the selected compounds were screened for anticonvulsant activity by supramaximal electric shock method. Some compounds exhibited inhibition of seizure relative to the control with the significant decrease in duration of extension phase at dose of 25 mg/kg^93^.

Several 4-alkyl-1,2,4-triazole-3-thione derivatives were prepared by Plech et al. as new anticonvulsant agents. Significant anticonvulsant activity was obtained for these compounds in the maximal electroshock-induced seizure (MES) test. 4-Butyl-5–(3-chlorophenyl)-2,4-dihydro-3*H*-1,2,4-triazole-3-thione (**68**, Figure 11) showed the strongest anticonvulsant activity after 15 min *via* systemic administration with ED_50_ value of 38.5 mg/kg in MES test. Chromatographic tests allow one to state that the lack of permeability through the blood-brain barrier was the reason for the lack of activity of some compounds in this series^94^.

Several bis-1,2,4-triazoles derivatives (**69**, Figure 11) were prepared by Kumudha et al. to evaluate their anticonvulsant. Both MES method and PTZ model confirmed the moderate to good anticonvulsant activity of these compounds. No neurotoxicity was found at the dose of 100 mg/kg^95^.

Several 1,2,4-triazol-3-thiols (**70**, Figure 11) containing 5-amino-1,3,4-thiadiazole were prepared by Kumudha’s group to evaluate their anticonvulsant, CNS depressant activities and neurotoxicity. The target compounds showed excellent anticonvulsant activity in both MES and PTZ tests, which significantly reduced the duration of tonic hind limb extensor in the MES model and significantly prolonged the onset of seizure against convulsions induced by PTZ^96^.

**Figure 12.**
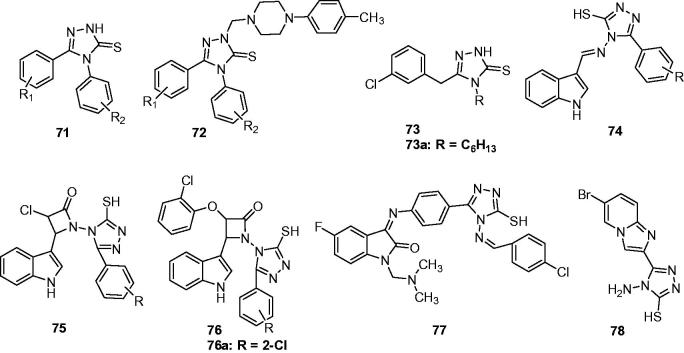
Substituted triazolthiones (**71**–**78**).

Several 1,2,4-triazole-3-thione derivatives (**71** and **72**, Figure 12) were prepared by Plech’s group to evaluate their anticonvulsant activity. Rapid onset of action and long lasting effect of these compounds were found in the MES model. Addition, a SAR was obtained that the anticonvulsant activity request at least one of the phenyl rings attached to 1,2,4-triazole nucleus, and the phenyl had a substituent at the para position. The radio-ligand binding assay (with benzodiazepine binding sites and GABAa receptors complex) suggested that a direct involvement of GABAA receptors or bind with BDZ-binding sites was excluded for their molecular mechanism^97^.

To obtain more SARs and higher activity of anticonvulsant agents, Plech’s group prepared another 1,2,4-triazole-3-thione derivatives (**73**, Figure 12) via the cyclisation of 1-acyl-4-alkylthiosemicarbazides. Taking into the account of the activity, toxicity and the time-course changes of ED_50_, **73a** (Figure 12) was proposed as the best tolerated and the most promising potential drug candidate (ED_50_ = 72.1 mg/kg and TD_50_ > 1000 mg/kg at 15 min interval). Like the previous work, the interactions (direct or allosteric) with GABAA receptor complex and/or the affinity to benzodiazepine (BDZ) binding sites was excluded for its molecular mechanism^98^.

Several indole derivatives (**74**, **75**, and **76**, Figure 12) containing triazolethione were synthesised and screened for their anticonvulsant activity by MES test in rats. All the compounds showed the protection against MES induced seizures in varying degrees. Among them, compound **76a** (Figure 12) substituted with 2-chloro showed interesting anticonvulsant activity with a considerable percentage of protection (90%) against MES induced seizure at the dose of 15 mg/kg, which exhibited better anticonvulsant activity than standard drug. The newly synthesised compounds were also tested for approximate lethal dose (ALD_50_) and were found to exhibit a higher value of ALD_50_ i.e. more than 1000 mg/kg, *i.p.* except compound **76a** which exhibited ALD_50_ of more than 2000 (maximum dose tested), indicating the safer nature of these compounds^99^.

Prakash et al. synthesised some isatin containing congeners of 1,2,4-triazole Schiff and Mannich bases with a view to explore their potency as better anticonvulsant agents. All the synthesised compounds were screened for its anti-epileptic activity by MES and scPTZ methods using phenytoin and ethosuximide as standards. The results showed that some of the synthesised compounds were exhibited significant activity. The most active was **77** (Figure 12) that revealed protection in the electrically induced seizures at a dose of 30 mg/kg at 0.5 h and 4 h after *i.p.* administration, respectively. This molecule provided also protection in the scPTZ at a dose of 100 mg/kg in 0.5 h and 300 mg/kg at 4.0 h time intervals^100^.

Ulloora et al. reported the facile synthesis and anticonvulsant study of new imidazo[1,2-a] pyridines carrying 1,2,4-triazole moieties. Most of the compounds displayed remarkable anticonvulsant properties at 20, 40, or 100 mg/kg. Compound **78** (Figure 12) carrying 4-amino-1,2,4-triazole moiety displayed the highest activity (100% protection at 20 mg/kg) at both the intervals (0.5 and 4 h) indicating its fast onset and long duration of action^101^.

## 5. Fused-triazoles

**Scheme 4.**
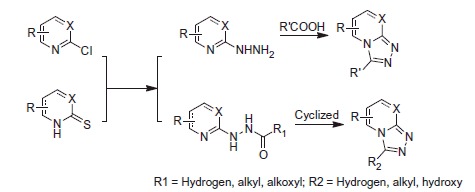
The classical preparation method of fused-triazoles.

There are various methods for synthesis of fused-triazoles. The most general method to introduce triazole ring for the fused-triazoles is cyclisation of halogenated heterocyclic or thio-substituted heterocyclic compounds with hydrazines (Scheme 4)^102^^,^^103^. Many fused-triazoles exhibited higher anticonvulsant activity because of the raise of the electronic cloud density in the triazole ring.

### 5.1 Bicyclic fused-triazoles

#### 5.1.1 Triazolopyrimidines, triazolopyridazines, triazolopyridines, and triazolopyrazines

**Figure 13.**
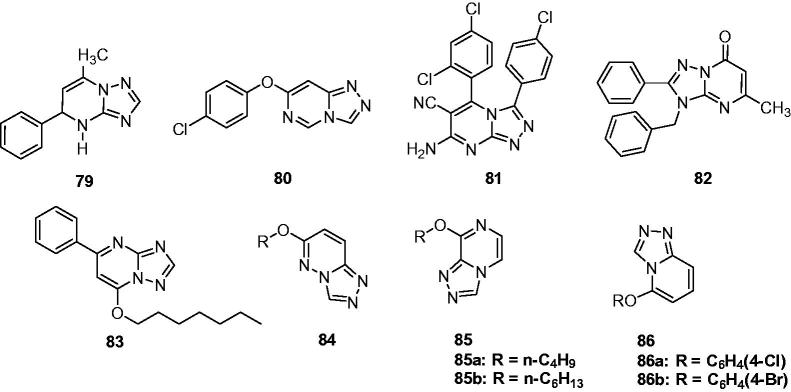
Triazolopyrimidines, triazolopyridazines, triazolopyridines, and triazolopyrazines (**79**–**86**).

Several fused triazolopyrimidine derivatives were synthesised by Said et al., and their anticonvulsant activity against yohimbine-induced clonic seizures in mice were evaluated. Compound **79** (Figure 13) was the most active one with ED_50_ of 10 mg/kg in this model, which displayed more potent than carbamazepine (28 mg/kg)^102^.

As part of the program finding new anticonvulsant agents in heterocyclic fused triazoles, several [1,2,4]triazolo[4,3-f]pyrimidine derivatives were designed and synthesised by Guan and coworkers. The anticonvulsant activity screens showed that the compound **80** (Figure 13) was the most active agent with an ED_50_ value of 34.7 mg/kg, a TD_50_ of 262.9 mg/kg, and PI value of 7.6^103^.

A series of [1,2,4]triazolo[4,3-a]pyrimidine derivatives were synthesised in good yields by the microwave-assisted one-pot protocol in very short reaction time. A prediction of anticonvulsant activities applying computer program PASS indicated that compound **81** (Figure 13) was a promising anticonvulsant agent^104^.

Recently, several novel BZD agonists were designed and synthesised based on the pharmacophore/receptor model of BZD binding site of GABAA receptor. Their affinity *in vitro* for the central BZD receptor was determined. Most of the novel compounds had better affinity for the BZD site of action on GABAA receptor complex than diazepam. Compound **82** (Figure 13) with the best affinity in radio-ligand receptor binding assay (*K*_i_ = 0.42 nM and IC_50_ = 0.68 nM) was selected as candidate for *in vivo* evaluation. This compound showed significant hypnotic activity and anticonvulsant effect [ED_50_ = 15.01 (MES model), 56.57 (PTZ model)] with no impairment on learning and memory performance in mouse. The pharmacological effects of the compound **82** were antagonised by flumazenil, a BZD antagonist, which confirms the involvement of BZD receptors in the biological effects of the novel ligand^105^.

Another triazolopyrimidines were synthesised by Jiang et al. through incorporating triazole moiety into the pyrimidine ring, which were expected to have the synergistic effect in dealing with the epilepsy. Their anticonvulsant activities against MES induced seizures were measured in mice. Carbamazepine and valproate were considered as positive control drugs with anticonvulsant effects [ED_50_ = 11.8 and 272 mg/kg]. Amongst these compounds, compound **83** (Figure 13) showed potent anticonvulsant activity with ED_50_ 84.9 mg/kg, which was weaker than carbamazepine, but better than valproate^106^.

Guan et al. reported a series of 6-alkoxy-[1,2,4]triazolo[4,3-b]pyridazine derivatives (**84**, Figure 13) as anticonvulsant agents. In MES model, compound **84a** (Figure 13) with R = C_6_H_3_ (2,4-2Cl) was the most active agents with the lowest toxicity with an ED_50_ of 17.3 mg/kg and TD_50_ of 380.3 mg/kg, which give a higher PI value than the reference drugs carbamazepine and phenobarbital (22 VS 8.1 and 3.2)^107^.

Following the above work, a series of new 8-alkoxy-[1,2,4]triazolo[4,3-a]pyrazine derivatives (**85**, Figure 13) were synthesised as anticonvulsant agents. The most promising compound **85a** with butyl substituent and compound **85b** with hexyl substituent (Figure 13) showed an ED_50_ of 44 and 35.3 mg/kg in MES test and had protective index value of 3.2 and 4.8, respectively^108^.

Meanwhile, the synthesis and anticonvulsant evaluation of a series of 5-alkoxy-[1,2,4] triazolo[4,3-a]pyridines (**86**, Figure 13) were carried out by Guan and coworkers. Based on the MES test and rotarod test, compounds **86a** (Figure 13) and **86b** (Figure 13) showed promising anticonvulsant activity with an ED_50_ of 13.2 and 15.8 mg/kg and had a protective index value of 4.8 and 6.9, respectively^109^.

#### 5.1.2 Triazolothiazoles, triazolothiadiazines, triazolothiadiazoles, and triazolothiazepines

**Figure 14.**
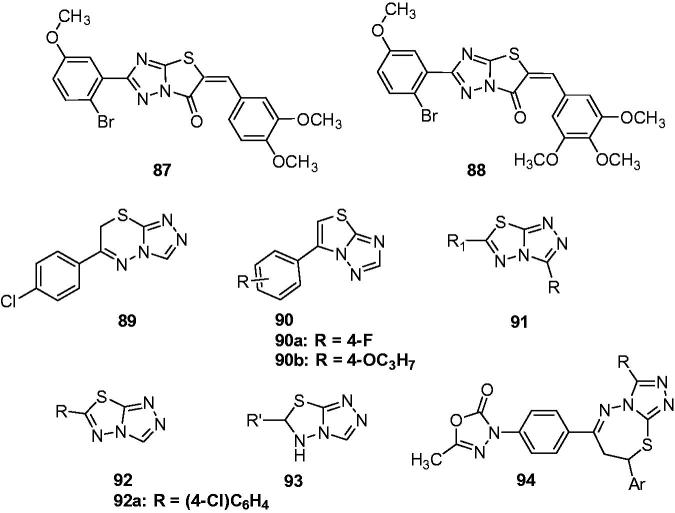
Triazolothiazoles, triazolothiadiazines, triazolothiadiazoles, and triazolothiazepines (**87**–**94**).

Narayana and Vijaya Raj reported a smooth synthesis of a series of thiazolotriazolones and the anticonvulsant activity screening of part compounds. Compounds **87** and **88** (Figure 14) exhibit promising anticonvulsant activity with a significant reduction of the duration of seizure in PTZ test at the dose of 4 mg/kg^110^.

Various triazolothiadiazines were designed and synthesised by Quan’s team. All the compounds were evaluated for their anticonvulsant activity against MES-induced seizures. Among of which, compound **89** (Figure 14) was the most promising compound with an ED_50_ value of 40.9 mg/kg and a PI value of 6.5. The SARs indicated that any substituent of R’ would decrease the anticonvulsant activity^111^.

In the next work, numbers of triazolotriazoles **90** (Figure 14) were synthesised and tested for their anticonvulsant activities using the MES and PTZ screens. In the MES test, compound **90a** (Figure 14) was found to be the most active compound, which gave an ED_50_ of 49.1 mg/kg. In the PTZ model the most active compound of tested compounds was **90b** (Figure 14), which showed an ED_50_ of 63.4 mg/kg. In this work, compounds holding big lipophilicity (CLoP > 3.9) did not show anticonvulsant activity, which was explained by the interrupting of the absorption and distribution^112^.

A series of 3,6-disubstituted-1,2,4-triazolo-1,3,4-thiadiazole derivatives (**91**, Figure 14) were synthesised and evaluated for their anticonvulsant activity and neurotoxicity. Compounds with bromophenyl group on the thiadiazole showed potent anti-MES activity comparable to that of standard drugs phenytoin and carbamazepine, which indicated that halosubstituted aryl (bromophenyl) in position 6 of the triazolothiadiazole ring was beneficial for the activity^113^.

6-Substituted-[1,2,4]triazolo[3,4-b][1,3,4]thiadiazole derivatives (**92**, Figure 14) and their partially hydrogenated products 5,6-dihydro-6-substituted-[1,2,4]triazolo[3,4-b][1,3,4]thiadiazole derivatives (**93**, Figure 14) were prepared as anticonvulsant agents by Deng et al. The MES test results demonstrated that the hydrogenation of the thiadiazole ring decreased the anti-MES activity significantly. Compound **92a** (Figure 14) with 4-chlorophenyl substituent emerged as the most promising candidate based on its favourable ED_50_ value of 23.7 mg/kg and PI value of 10.8. In addition, the potency of compound **92a** against seizures induced by PTZ, 3-mercaptopropionic acid, and bicuculline suggested that compound **92a** displayed broad-spectrum activity, and it might exert its anticonvulsant activity through affecting the GABAergic system^114^.

**Scheme 5.**
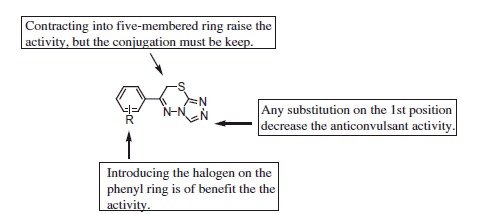
The structure-activity diagram of the triazolothiadiazines.

Comparing the anticonvulsant activity of compounds **89**, **91**, **92**, and **93**, it can be found that the conjugation between the heterocycle and triazole is very important for their anticonvulsant activity. The SAR information was described as below (Scheme 5).

Kamble and Sudha reported an efficient synthesis of pharmacologically active derivatives of 1,3,4-oxadiazoles (**94**, Figure 14), in which some triazole-containing derivatives were included. Several triazole-containing derivatives were found to possess very good activity against MES induced convulsions in rats when compared to that of standard phenyntoin^115^.

### 5.2 Tricyclic fused-triazoles

#### 5.2.1 Triazoloquinolines

**Figure 15.**
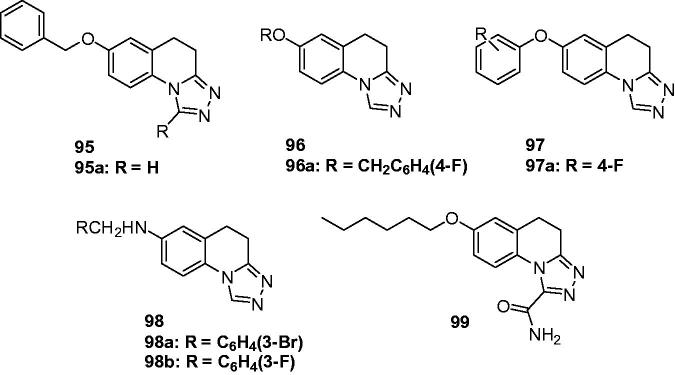
Triazoloquinolines (**95**–**99**) with anticonvulsant activity.

In 2005, the team of Quan launched a study for new anticonvulsant agents by incorporating triazole with various heterocyclic. Based on the weak activity of 6-benzyloxy-3,4-dihydro-1Hquinoline-2-one, a series of 1-substituted-7-benzyloxy-4,5-dihydro-[1,2,4]triazolo[4,3-a]quinolines (**95**, Figure 15) were prepared by incorporating triazole with 6-benzyloxy-3,4-dihydro-1*H*-quinolinone for more potent anticonvulsant activity. The results indicated that the introduction of triazole increased the activity, but any substitutions at the first position decreased the anticonvulsant activity markedly. Compound **95a** with H in the 1-position was the most active one with an ED_50_ of 17.3 and 24 mg/kg, respectively in the MES and PTZ model tests^116^.

As a continuation of the above work, 7-alkoxyl-4,5-dihydro-[1,2,4]triazolo[4,3-a]quinoline derivatives (**96**, Figure 15) were then prepared. Most of them displayed potent anticonvulsant activity against the MES and scPTZ-induced seizures. The SAR indicated that the lengthening of the alkyl chain at position 7 influenced the anticonvulsant activity obviously, in which the n-hexyl-substituted compound was found to be the most active. Among all the compounds in this series, **96a** (Figure 15) was found to be the most promising one with ED_50_ of 11.8, and 6.7 mg/kg in the MES and PTZ model tests, respectively, although the neurotoxicity was also followed with a TD_50_ of 54.5 mg/kg in the rotarod test^117^.

To obtain broader SARs of triazoloquinolines and get higher activity anticonvulsants, several 7-aryloxyl-4,5-dihydro-[1,2,4]triazolo[4,3-a]quinolines (**97**, Figure 15) were prepared by Quan et al. All compounds in this series exhibited potent anti-MES and anti-PTZ activities with the ED_50_ range of 6.8–50.9 mg/kg. The introduction of halogen (especially the fluorine atom) into the side-chain benzene ring significantly increased the anti-MES activity, giving the most promising one **97a** (Figure 15) with an ED_50_ of 6.8 mg/kg in the MES test^118^.

In order to obtain a novel anticonvulsant agent having more potency and lower neurotoxicity, a series of 7-substituted-benzylamino-4, 5-dihydro-[1,2,4]triazolo[4, 3-a]quinoline derivatives (**98**, Figure 15) was synthesised and evaluated for their anticonvulsant activity. Compound **98a** (Figure 15) was the most effective one in PTZ test with an ED_50_ of 5. 0 mg/kg and the PI of 20.7, which was also safer than the reference drugs. In MES test, compound **98b** (Figure 15) was the most promising one with an ED_50_ of 15.3 mg/kg and the PI of 7.2^119^.

A number of 1-aminocarbonyl-triazolo[4,3-a]quinoline derivatives was prepared by Quan’s team via linking the key group of anticonvulsant agents, i.e. carboxamide group to the triazolo[4,3-a]quinoline. The anticonvulsant effect and neurotoxicity of the compounds was evaluated with MES test and rotarod tests in mice. The SAR indicated that alkyl substitutes was good for activity than the benzyl substitutes. Compound **99** (Figure 15) was the most active one with an ED_50_ of 30.1 mg/kg, and had the lowest toxicity with an TD_50_ of 286 mg/kg, which gave the greater PI value of 9.5 than the reference drug carbamazepine (PI = 6.0)^120^.

**Figure 16.**
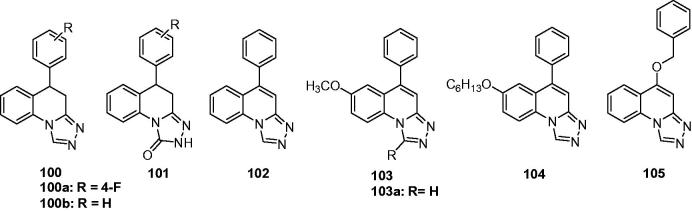
Triazoloquinolines (**100**–**105**) with anticonvulsant activity.

A series of substituted 1,2,4-triazolo[4,3-a]-quinoline (**100**, Figure 16) and 1,2,4-triazolo[4,3-a]-quinolinone derivatives (**101**, Figure 16) were designed and synthesised to meet the structural requirements essential for anticonvulsant properties. The results revealed that the introduction of triazole had increased anticonvulsant effects compared to the parental compounds quinolinones. However, the introduction of triazolone give the 1,2,4-triazolo[4,3-a]-quinolinone derivatives **101** with no anticonvulsant effects even under the high dose of 300 mg/kg. Among this work, compound **100a** (Figure 16), showed the strongest anticonvulsant effect with ED_50_ of 27.4 mg/kg and 22.0 mg/kg in the anti-MES and anti-PTZ test, respectively^121^.

In order to obtain compounds with better anticonvulsant activity, structural modification was made on the compounds **100** i.e. the introduction of a double bond into the fourth and fifth positions to give the compound **102** (Figure 16). A further modification was made via introducing a methoxy group in the seventh position, and then an alkyl (or aryl) in the first position of the 1,2,4-triazolo[4,3-a]-quinolines to give the compounds **103** (Figure 16). Compound **102** (ED_50_ = 28.4 mg/kg) showed higher activity than compound **100 b** (R = H, ED_50_ = 54.8 mg/kg) in MES test. While in the series of **103**, compound **103a** (Figure 16), with ED_50_ of 9.2 mg/kg, was the most promising one with the anticonvulsant activity comparable to that of the reference drug phenytoin (ED_50_ = 9.9 mg/kg) in MES test. In addition, compound **103a** showed lower neurotoxicity than phenytoin with a TD_50_ of 151 mg/kg, and gave a PI value of 16.6 in the MES test, which was higher than the PI value of phenytoin. Compound **103a** also antagonised PTZ and isoniazid-induced seizures with an ED_50_ of 21.1 mg/kg and 83.3 mg/kg, respectively, which suggested that compound **103a** might exert anticonvulsant activity by impacting GABA-ergic neurotransmission and the glycine system^122^.

A series of new derivatives of compound **102** was prepared to obtain more SARs of [1,2,4]-triazolo[4,3-a]quinolines and better anticonvulsant compounds. This study gave a potent compound **104** (QUAN-0806, Figure 16) with an ED_50_ value of 6.5 mg/kg in MES test and a protective index value of 35.1, which was much higher than the PI of the reference drug phenytoin (PI = 6.9)^123^. The oral ED_50_ of QUAN-0806 was 44.7 mg/kg in MES test in mice, and the oral TD_50_ of that was bigger than 2000 mg/kg. The anticonvulsant activity of QUAN-0806 was further investigated against seizures induced by PTZ, 3-MPA, ISO, and STRYC to investigate the possible mechanisms behind this activity. The results resealed that QUAN-0806 was effective against seizures induced by PTZ, ISO, and 3-MPA with ED_50_ values of 25.0, 39.4, and 19.7 mg/kg, respectively^124^.

In the previous work, the introduction of the double bond was considered as beneficial based on the improvement of the anticonvulsant activity. So, in the next work, a series of 5-alkoxy-[1,2,4]triazolo[4,3-a]quinoline derivatives were prepared by Quan’s team. The hypothesis is that the electron density at triazole ring will be increased because of the lone pair electrons of oxygen atom of 5-alkoxy via the conjugation effect, so as the affinity of compounds to the receptor will be increased. The pharmacological results revealed that most of the compounds showed remarkable anticonvulsant activity, but the serious toxicity also followed. Taking consideration of the safety, compound **105** (Figure 16) was considered as the most promising one with ED_50_ of 22.8, TD_50_ of 273.9, and PI value of 12.0. Additional, compound **105** produced significant protection activity against seizures induced by PTZ, 3-MP, thiosemicarbazide and isoniazid, but not strychnine suggested that the compound **105** might have effects on GABAergic neurotransmission and activate GAD or inhibit GABA-T in the brain^125^.

#### 5.2.2 Triazolo-quinazoline, phthalazine, and quinoxaline derivatives

**Figure 17.**
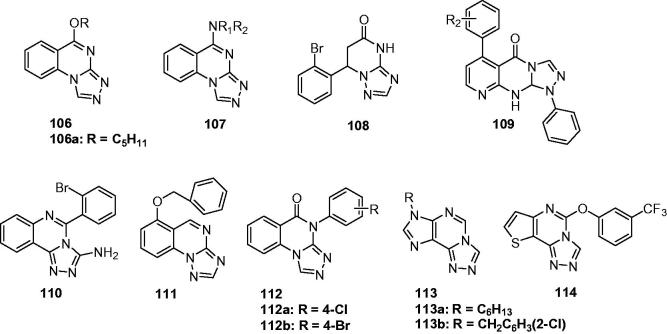
Triazoloquinazolines (**106**–**114**) with anticonvulsant activity.

Two series of 5-substituted[1,2,4]triazolo[4,3-a]quinazolines (**106** and **107**, Figure 17) were synthesised to screen their anticonvulsant activity in MES test. The pharmacology results showed that compound **106a** (Figure 17) with pentyl group was the most potent with ED_50_ value of 19.7 m g/kg and PI value of 6.2. It is interesting that alkyl amino substituted derivatives (**107**) exhibited low activity and high toxicity. It suggested the avoiding of this group in the later design of anticonvulsants^126^.

A group of 7-(substituted-phenyl)-6,7-dihydro-[1,2,4]triazolo[1,5-a]pyrimidin-5(4*H*)-ones were prepared as anticonvulsant agents by Deng et al. Most of the synthesised compounds exhibited potent anticonvulsant activities in the MES test. The most promising compound (**108**, Figure 17) showed significant anticonvulsant activity in MES test with ED_50_ value of 19.7 mg/kg and low toxicity in rotarod test with TD_50_ value of 684.7 mg/kg, which gave a high PI value of 34.8^127^.

Kawade et al. synthesised another series of triazolopyrimidine-5(4*H*)-ones (**109**, Figure 17) to screen their anticonvulsant activity. The results indicated that all compounds showed activity with ED_50_ range from 152 to 667 mg/kg in MES test. Furthermore, it has been found that the ED_50_ and TD_50_ values of test compounds increase significantly at *t* = 4 h, when compared to *t* = 30 min, in contrast to the standard Phenytoin^128^.

In the work of Zheng et al. for preparing same quinazolintriazoles as anticonvulsant agents, 5-(2-bromophenyl)-[1,2,4]triazolo[4,3-c]quinazolin-3-amine (**110**, Figure 17) was obtained, which showed an ED_50_ value of 27.4 mg/kg and a TD_50_ value of 157.8 in the MES and rotarod test, respectively. This work found that the introducing of NH_2_ to the triazole was beneficial to the anticomvulsant activity for these quinazolinetriazoles^129^.

In another work, a series of novel 6-alkyoxyl[1,2,4]triazolo[1,5-a]quinazoline derivatives were synthesised and evaluated for anticonvulsant activity using MES tests. The pharmacological results showed that some of the compounds displayed positive anticonvulsant activity. Among them, compound **111** (Figure 17) was the most active compound with an ED_50_ value of 78.9 mg/kg and a PI value of 9.0^130^.

As the continue of the above works, another triazolopyrimidine derivatives (**112**, Figure 17) were synthesised by Quan’s team and evaluated for their anticonvulsant activity and neurotoxicity. In the MES test, among the compounds studied, **112a** (Figure 17) and **112b** (Figure 17) showed potent anti-MES activity with an ED_50_ of 27.4 and 26.9 mg/kg, and wide margins of safety with PI value of above 25.5 and 26.9, respectively. The two compounds were also found to have potent activity against seizures induced by PTZ and bicuculline at 50 mg/kg in mice^131^.

Several [1,2,4]triazolo[4,3-g]purines (**113**, Figure 17) were also prepared by Quan’s group to more effective AEDs. Compounds **113a** and **113b** were considered as the most promising compounds with an ED_50_ of 51.2 and 51.9 mg/kg in MES test, respectively^46^. Meanwhile, a series of triazolopyrimidines were prepared and evaluated for their anticonvulsant activities. The SARs analysis revealed that the most effective structural motif involves a substituted phenol, especially when substituted with a single chlorine, fluorine or trifluoromethyl group (at the meta-position), or two chlorine atoms. The most active compound in this series was **114** (Figure 17) with ED_50_ values of 11.5 mg/kg (MES) and 58.9 mg/kg (scPTZ)^132^.

**Figure 18.**
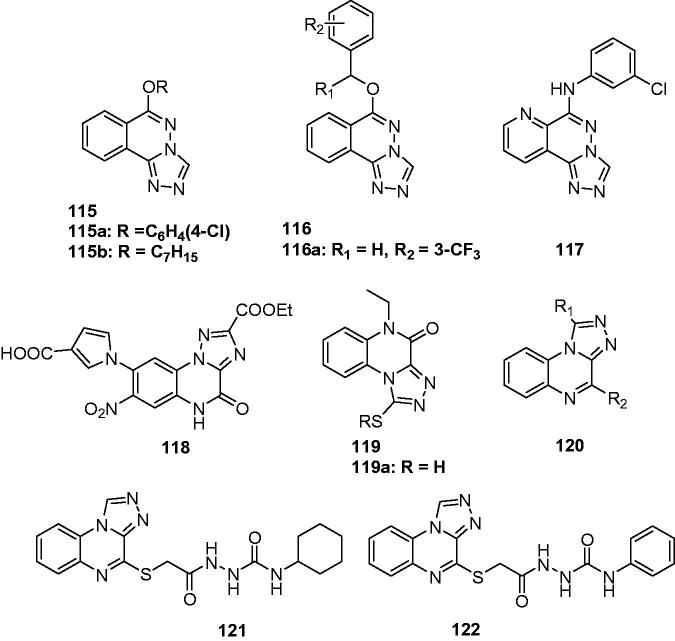
Triazolophthalazine and quinoxalines (**115**–**122**) with anticonvulsant activity.

A new series of 6-alkoxy-[1,2,4]triazolo[3,4-a]phthalazines (**115**, Figure 18) were synthesised as an isostere of compounds **106**, which held potent anticonvulsant activity as shown above. In this series, the most promising compounds **115a** (Figure 18) and **115b** (Figure 18) showed a median effective dose of 7.1 and 11.0 mg/kg, and had protective index value of 5.2 and 8.0, respectively. The two compounds were further found to have potent activity against seizures induced by pentylenetetrazole, isoniazid, thiosemicarbazide, 3-mercaptopropionic acid but not seizures induced by strychnine, indicating that the two compounds might function by enhancing gamma-aminobutyric acid neurotransmission^133^.

Bian et al. described the synthesis and anticonvulsant activity evaluation of 6-substituted-[1,2,4]triazolo[3,4-a]phthalazines (**116**, Figure 18). Most of the synthesised compounds exhibited potent anticonvulsant activities in the maximal electroshock test (MES). The most promising compound **116a** (Figure 18) showed significant anticonvulsant activity in MES test with ED_50_ value of 9.3 mg/kg. It displayed a wide margin of safety with protective index much higher than the standard drug Carbamazepine (91.7 versus 6.4)^134^.

A series of 6-substituted-pyrido[3,2-*d*]pyridazine derivatives containing triazole was synthesised as the isosteres of **115**. Their anticonvulsant activities were evaluated by the MES and their neurotoxicity were measured by the rotarod test. The results demonstrated that compound **117** (Figure 18) was the most potent anticonvulsant, with ED_50_ value of 13.6 mg/kg and protective index values of 7.2 in the MES test^135^.

Catarzi et al. reported 4,5-dihydro-4-oxo-1,2,4-triazolo[1,5-a]quinoxaline-2-carboxylates (TQXs) and 3-hydroxy-quinazoline-2,4-diones (QZs) as new AMPA receptor antagonists. High binding affinity and selectivity for the AMPA receptor were obtained in the [3*H*]-6-cyano-7-nitroquinoxaline-2,3-dione ([3*H*]-CNQX) binding assay. Compound **118** (Figure 18) was the most potent and selective AMPA receptor antagonists. It displayed its ability to prevent sound-induced seizures in DBA/2 mice with 83% protection against tonic seizures at 30 mg/kg^136^.

A series of 1,2,4-triazolo(4,3-a)quinoxalin-4-*5H*-ones **119** (Figure 18) were prepared as AMPA receptor antagonists. Their anticonvulsant activity was confirmed by the protection activity against PTZ induced seizures. Compound **119a** (Figure 18) was the most potent one with a ED_50_ of 12.5 mg/kg in PTZ induced seizures model. There was a strong correlation between the results of the anticonvulsant activity and molecular modelling, which suggested that the AMPA receptor antagonism was involved in their anticonvulsant activity^137^.

1,4-Disubstituted[1,2,4]triazolo[4,3-a]quinoxalines (**120**, Figure 18) were prepared by Wagle et al. as potent anticonvulsants. Some of the compounds exhibited 100% protection for the animals against PTZ induced convulsions at the dose of 10 mg/kg. The SARs study revealed that compounds bearing CF_3_, H or CH_3_ group in position-1 and 4-fluorophenyl moiety or 4-methoxyphenyl substituents at C-4 of these compounds have shown good anticonvulsant activity in comparison with standard drug diazepam^138^.

Based on the promising anticonvulsant activity of [1,2,4]triazolo[4,3-a]quinoxaline moiety, other [1,2,4]Triazolo[4,3-a]quinoxaline derivatives were prepared by Alswah et al. as novel anticonvulsant agents. Among those compounds, two of them (**121** and **122**, Figure 18) showed the best anticonvulsant activities with an ED_50_ value of 30 mg/kg in the PTZ model^139^.

#### 5.2.3 Triazolobenzothiazines and triazolobenzothiazoles derivatives

**Figure 19.**
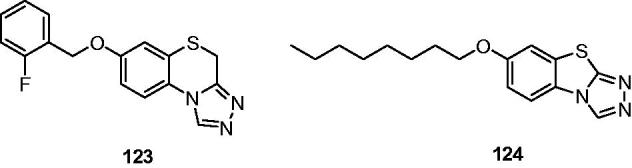
Triazolobenzothiazines and triazolobenzothiazoles (**123** and **124**).

A series of triazolobenzothiazines was designed and synthesised by Quan’s team as an isostere of compounds **95**. Among these compounds, **123** (Figure 19) was the most active compound with an ED_50_ of 17.0 mg/kg and a protective index of 14.3 in the MES test^140^. Next, a series of 7-alkoxy-triazolo-[3,4-b]benzo[d]thiazoles was designed and synthesised as the ring contraction analogues of compounds **123** through removal of a CH_2_ in the compounds **123**, were anticipated to possess a better anticonvulsant activity. Among these compounds, **124** (Figure 19) was found to be the most potent compound with an ED_50_ of 8.0 mg/kg and a PI value of 15.0, possessing better anticonvulsant activity and higher safety than marketed drugs carbamazepine and phenytoin. The mechanism study of compound **124** showed that it displayed broad spectrum activity in several models, and it is likely to have several mechanisms of action (including inhibiting voltage-gated ion channels and GABAergic activity)^141^.

#### 5.2.4 Tricyclic fused-triazoles containing 7-membered ring

For the past three decades, five-atom heterocyclic fused benzodiazepine ring systems occupy a prominent place among drugs for treatment of CNS disorders^142^. The introduction of alprazolam and triazolam in epilepsy therapy has enhanced the interest in preparation triazole-benzodiazepine derivatives. Numerous analogues of alprazolam and triazolam along with their pharmacological profiles have been described.

**Figure 20.**
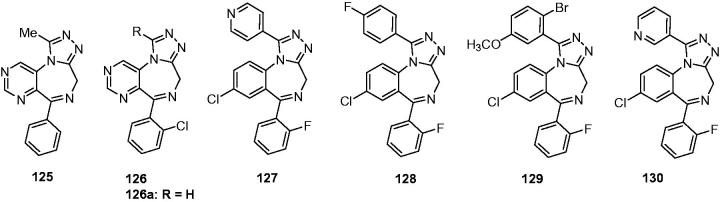
Triazolebenzodiazepines (**125**–**130**).

Based on the SAR for the triazole-benzodiazepine class of compounds that an electronegative substituent at C-7 is an essential requirement for anticonvulsant, Phillips et al. designed and synthesised a pyrimido[4,5-f][1,2,4]triazolo[4,3-a][1,4]diazepine derivative (**125**, Figure 20) and evaluated its anticonvulsant activity to find more potent anticonvulsant. Compound **125** showed moderately activity against MES-induced seizures with ED_50_ of 79.5 mg/kg, and high activity against PTZ-induced seizures with ED_50_ of 6.2 mg/kg. It was non-toxic under the dose of 150 mg/kg (TD_50_ >150 mg/kg *i.p.* in mice). The affinity for the benzodiazepine receptor was observed, but it was weaker than the reference drug clonazepam (IC_50_: 72 µM versus 0.00372 µM)^143^.

The synthesis and anticonvulsant activities of 5-(2-chlorophenyl)-7*H*-pyrido[4,3-f]-[1,2,4]triazolo[4,3-a][1,4]diazepines (**126**, Figure 20) were reported by Knaus’ team. Their anticonvulsant activities were determined by the Antiepileptic Drug Development Program using the two common models: PTZ and MES models. All the compounds in this series showed potent anti-PTZ activity, but not anti-MES activity. The compound **126a** (Figure 20) was the most promising one, which hold the ED_50_ of 0.069 mg/kg in the PTZ model at 15 min^144^.

Based on the SARs of triazole-benzodiazepines that the electron withdrawing substituents such as fluoro, chloro, and bromo at C-8 and C′-2 confer high anticonvulsant activity, novel 8-chloro-6-(2-fluorophenyl)-1-(aryl)-4*H*-[1,2,4]triazolo[4,3-a][1,4]benzodiazepines were prepared and screened their anticonvulsant activity by Narayana’s team. Four of the tested compounds **127**, **128**, **129**, and **130** (Figure 20) exhibited excellent anticonvulsant activity in comparison with standard drug diazepam with complete protection against PTZ-induced seizures at the dose of 4 mg/kg, and a reduction of the duration of tonic hind limb extensor in MES test at the same dose^145^.

**Figure 21.**
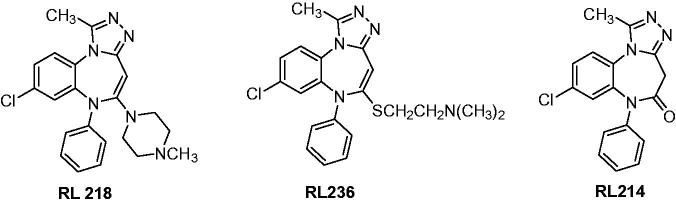
The structures of **RL 218** and **RL 236** and their hydrolyzate **RL 214**.

Caccia et al. studied the pharmacokinetics of two benzodiazepine compounds **RL 218** and **RL 236** (Figure 21). Their metabolite **RL 214** (Figure 21) along with themselves was evaluated for the anticonvulsant activity against pentylenetetrazole-induced lethal convulsions in mice. Compounds **RL 218** and **RL 236** were active against pentylenetetrazole-induced lethal convulsions in mice only when administered orally, with ED_50_ of 31.9, and 60.7 mg/kg, respectively. **RL 214** were comparable or preferable when compared to **RL 218** and **RL 236** after taking this compound orally, with an ED_50_ of 29.3 mg/kg. **RL 214** but not **RL 218** or **RL 236** had *in vitro* affinity for brain benzodiazepine receptors. Which indicated that the anticonvulsant activity of **RL 218** and **RL 236** in mice were from their in-vivo active metabolite **RL 214**^146^.

**Figure 22.**
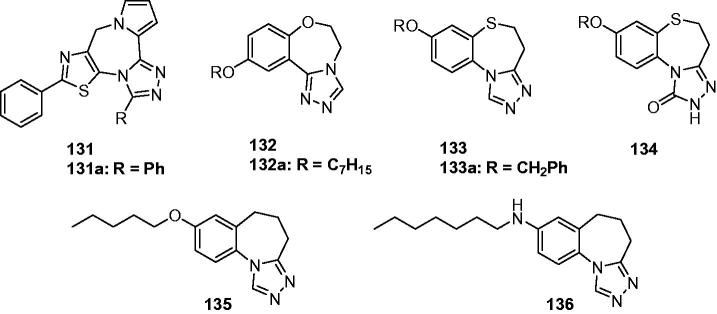
Tricyclic fused-triazoles containing 7-membered ring (**131**–**136**).

Shekarchi et al. reported the synthesis of 7-phenyl-5*H*-thiazolo[5,4-*e*][1,3,4]triazolo[5,1-c]pyrrolo[1,2-*a*][1,4]diazepines (**131**, Figure 22) and anticonvulsant activity against PTZ-induced seizures in mice. Intraperitoneal injections of different doses (12.5, 25, and 50 mg/kg) of these compounds decreased PTZ-induced seizure significantly in a dose-dependent manner. Pre-treatment of animals with flumazenile (as a benzodiazepine, BDZ receptor antagonist) decreased but not completely the anticonvulsant activity of compound **131a** (Figure 22), which suggested that the BZD receptors system may be partly involved in the anticonvulsant activity of the tested compounds^147^.

A series of 10-alkoxy-5,6-dihydro-triazolo[4,3-d]benzo[f][1,4]oxazepines (**132**, Figure 22) were designed and synthesised by Deng et al., as the ring enlargement analogues of compounds **95**. Among of which, compound **132a** was found to possess better anticonvulsant activity and higher safety than marketed drugs carbamazepine and phenytoin with an ED_50_ value of 6.9 mg/kg a PI value of 9.5. To explain the possible mechanism of anticonvulsant activity, compound **132a** was tested in pentylenetetrazole, isoniazid, thiosemicarbazide, 3-mercaptopropionic acid, and Bicuculline induced seizures tests. The results suggested that compound **132a** exerts anticonvulsant activity through GABA-mediated mechanism^148^.

Based on the potent anticonvulsant activity of compounds **123**, a series of ring-expanding products (**133**, Figure 22) and its carbonyl derivatives (**134**, Figure 22) were designed and synthesised. Their anticonvulsant activities and neurotoxicity were evaluated using the MES-induced seizure model and the rotarod assay in mice, respectively. Based on the results, compound **133a** containing benzyl group was the most active one with an ED_50_ of 20.7 mg/kg in MES test^149^.

In a study by Zhang et al. in 2012, a novel series of 8-alkoxy-5,6-dihydro-4*H*-benzo[f][1,2,4]triazolo[4,3-a]azepine derivatives were synthesised and screened by the MES test, scPTZ test, and rotarod test. Among the tested compounds, 8-pentyloxy-5,6-dihydro-4*H*-benzo[f][1,2,4]triazolo[4,3-a]azepine (**135**, Figure 22) with ED_50_ of 17.5 mg/kg in the MES test was more potent than phenobarbital (ED_50_ = 21.8 mg/kg) but slightly less than that of carbamazepine (ED_50_ = 8.8 mg/kg)^150^.

As a continuation of the previous work, a series of new 8-alkylamino-5,6-dihydro-4*H*-benzo[f][1,2,4]triazolo [4,3-a]azepine derivatives were synthesised and screened for their anticonvulsant activities by the MES test, scPTZ test, and rotarod test. The results of these tests showed that 8-heptylamino-5,6-dihydro-4*H*-benzo[f][1,2,4]triazolo[4,3-a]azepine (**136**, Figure 22) was the most promising compound, with an ED_50_ of 19.0 mg/kg, and PI value of 14.8 in the MES test, which is much higher than the PI value of the prototype antiepileptic drug carbamazepine (PI = 8.1), phenytoin (PI = 6.9), phenobarbital (PI = 3.2), and sodium valproate (PI = 1.6)^151^.

2-Amino-3-(3-hydroxy-5-methylisoxazol-4-yl)propionic acid (AMPA) receptor is one of Glutamate receptors (GluRs), which is an important target for the treatment of epilepsy. Several AMPA receptor antagonists have been reported in the literature and show promise in terms of their therapeutic potential for the prevention and treatment of a broad range of acute and chronic neurological diseases^152–155^.

**Figure 23.**
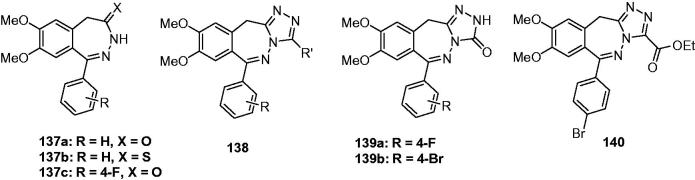
Tricyclic fused-triazoles containing 7-membered ring (**137**–**140**).

Gitto and his team strived to find new anticonvulsants based on the non-competitive AMPA-type glutamate receptor antagonists. Some of their previous publications^156–158^ reported chemical and biological studies of 1-aryl-3,5-dihydro-7,8-dimethoxy-4*H*-2,3-benzodiazepin-4-ones **137a** (Figure 23) and thiocarbonyl analogues **137b** (Figure 23), which had shown marked anticonvulsant activities in various seizure models. In order to obtained more promising AMPA antagonists with increased potency and selectivity, longer-lasting activity, and improved pharmacokinetic features, Gitto et al*. s*ynthesised a series of cyclo-functionalised 2,3-benzodiazepines: i.e. 11*H*-[1,2,4]triazolo[4,5-c][2,3]benzodiazepines **138** (Figure 23)^159^. However, it showed weaker anticonvulsant effects than the parent compounds **137a** and **137b**. To determine if the lower potency of the compounds **138** (Figure 23) was due to the cyclo-functionalisation of the diazepine ring, to the nature of the fused five-membered ring, or to the absence of the lactam moiety, a series of 11*H*-[1,2,4]triazolo[4,5-c][2,3]-benzodiazepin-3(2*H*)-ones **139** (Figure 23) was prepared subsequently. The compounds **139** were found to possess potent anticonvulsant effects against seizures induced both by means of auditory stimulation in DBA/2 mice and by pentylenetetrazole or maximal electroshock in Swiss mice. And comparison between the biological results of compounds **139** and **137** reveals that the introduction of the triazolone nucleus on the diazepine skeleton leads to compounds with comparable or higher anticonvulsant potency than the corresponding derivatives **137**. Taking compound **137c** (Figure 23) and **139a** (Figure 23) as an example, the ED_50_ of **137c** and **139a** against audiogenic seizures was 78.0 and 32.1 mg/kg, respectively. In addition, the property of compounds **139** that antagonising the AMPA-induced seizures, and the reverse of anticonvulsant activity by pre-treatment with aniracetam, suggested the involvement of AMPA receptors in their mechanism of action^160^.

As an attempt to further examine the role of cyclo-functionalisation and to gain more information from SAR studies, other compounds in the series of **139** were prepared by Gitto and his team in 2003. The promising one **139b** (Figure 23) was found with an ED_50_ of 3.65, 5.93, and 13.8 mg/kg against audiogenic seizures, MES induced seizure and PTZ induced seizure, respectively^161^.

As a continuation of the project, a series of 3-ethoxycarbonyl-11*H*-[1,2,4]triazolo[4,5-c][2,3]benzodiazepines were then synthesised, the potent anticonvulsant activities were also established against AMPA-induced seizures and audiogenic seizures in mice. Compound **140** (Figure 23) was the most active among the series with ED_50_ of 35.1 and 26.2 mg/kg against audiogenic seizures in clonic phase and tonic phase in mice, respectively^162^.

#### 5.3 Tetracyclic fused-triazoles

**Figure 24.**
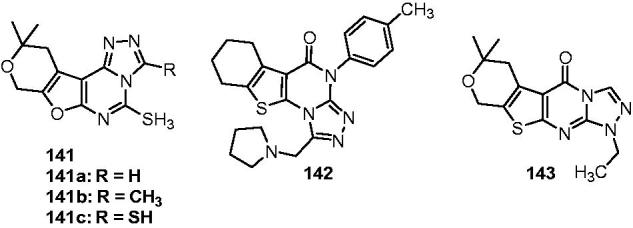
Tetracyclic fused-triazoles (**141**–**143**).

A series of furo[3,2-a]-I,2,4-triazolo-[4,3-a]prrimidines (**141**, Figure 24) were synthesis and tested for their anticonvulsant activity by Sc-PTZ model in mice. Compound **141a** (Figure 24) produced an anti-PTZ effect with 62 mg/kg. In contrast, compound **141b** and **141c** (Figure 24) possess no anticonvulsive in the preliminary test. This results suggested that the triazole was the indispensable part for the anticonvulsant activity of furo[3,2-a]-I,2,4-triazolo-[4,3-a]prrimidines^163^.

Thore et al. synthesised several triazolo [4, 3-a]tetrahydrobenzo (b) thieno [3,2-e] pyrimidine-5(4*H*)-ones. Some of them were found to protect 100% mice at the dose of 6–11 mg/kg at PTZ model. The most promising one was **142** (Figure 24), which completely protected the mice test against PTZ induced clonic convulsions at 6 mg/kg^164^.

As a continuation of the above work, several pyrano[4′,3′:4,5]thieno[3,2-e]triazolo[3,4-b]pyrimidine derivatives were synthesised and tested for their anticonvulsant activity. None of the compounds studied protected the experimental animals from the convulsion-inducing action of nicotine, arecoline, and MES. However, all drugs prevented PTZ induced convulsions. In the PTZ induced convulsions models, compound **143** (Figure 24) with the ED_50_ of 49 mg/kg, TD_50_ of 850 mg/kg and PI value of 17.3 was the most promising anticonvulsant agent^165^.

**Scheme 6.**
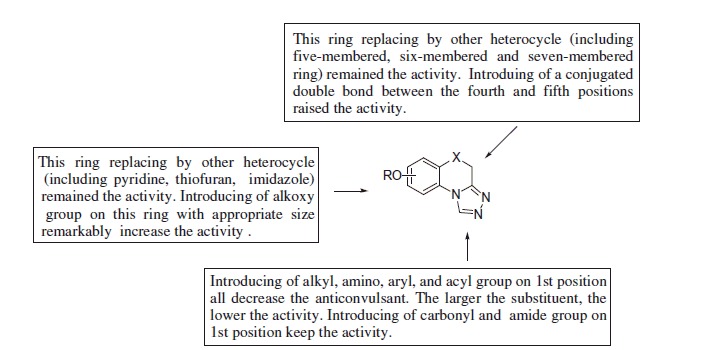
The structure-activity diagram of the triazoloquinolines.

In the fused-triazoles, triazoloquinolines was the most representative classes covering a large number of derivatives with potent anticonvulsant activity. Based on the available reports, the below SAR scheme could be drawn (Scheme 6).

## 6. Fused-triazolones

**Figure 25.**
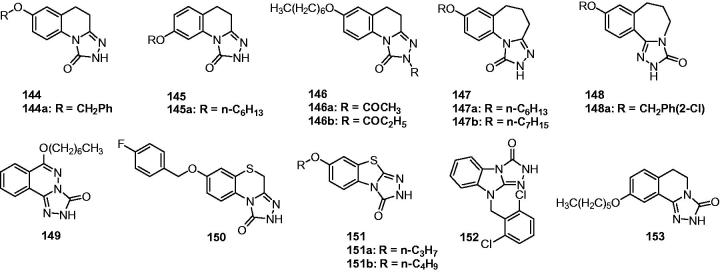
Fused-triazolones and fused-triazolthiones (**144**–**153**).

Based on the hypothesis that a triazolone compound may have higher affinity for the receptor due to its carbonyl group, and thus may have increased anticonvulsant activity, Quan’s team introduced the triazolone to the 3,4-dihydro-2(1*H*)-quinolines in 2006, which resulted two series of triazolonequinolines: 7-alkoxy-4,5-dihydro-[1,2,4]triazole[4,3-a]quinoline-1-ones (**144**, Figure 25) and 8-alkoxy-4,5-dihydro-[1,2,4]triazole[4,3-a]quinoline-1-ones (**145**, Figure 25). Their anticonvulsant activities were evaluated by the MES test and the sc-PTZ test, and their neurotoxicity were measured by the rotarod neurotoxicity test. Among the series of **144**, compound **144a** (Figure 25) was the most active along with the lowest toxicity. It showed an ED_50_ of 12.3 mg/kg in MES test, a TD_50_ of 547.5 mg/kg in rotarod test, and a protective index (PI) of 44.5, which is much greater than PI of the prototype drugs phenytoin, phenobarbital, carbamazepin, and valproate. Additionally, the median hypnotic dose (HD_50_) and median lethal dose (LD_50_) of 1204 mg/kg and >3000 mg/kg were also obtained for **144a**. Among the series **145**, 8-hexyloxy-4,5-dihydro-[1.2.4]triazole[4.3-a]quinoline-1-one **145a** (Figure 25) having ED_50_ values of 17.17 and 24.55 mg/kg and protective index values of 41.9 and 29.3 in the MES and sc-PTZ tests, respectively, was the most promising one. Compared to triazolo[4,3-a]quinolines, the triazole[4.3-a]quinolinone hold the considerable anticonvulsant activity, but much higher safety^166^^,^^167^.

In the next part of the above work, 2-substituted-7-heptyloxy-4,5-dihydro-[1,2,4]triazolo[4,3-a]quinoline-1(2*H*)-ones (**146**, Figure 25) were prepared to investigate the contribution of different acyl and alkyl groups at position 2 of the **146** to the anticonvulsant activity. The results revealed that neither alkylation nor acylation of compound **144** at the 2 position markedly decreased the anticonvulsant activities except small acyl group. Compound **146a** (Figure 25) was the most active one with an ED_50_ values of 7.2 mg/kg, and PI value of 12.2, which exhibited more potent anticonvulsant activity than compound **144a** and the reference drugs phenytoin and carbamazepine. Compound **146 b** (Figure 25) was the safest therapeutic compound with ED_50_ = 8.2 mg/kg, TD_50_ = 318.3 mg/kg and PI = 39.0^168^.

A series of novel 8-alkoxy-5,6-dihydro-4*H*-[1,2,4]triazolo[4,3-a][1]benzazepin-1-one derivatives (**147**, Figure 25) were synthesised as the ring enlargement product of **144**. Their anticonvulsant activities were evaluated by the MES test, PTZ test, and their neurotoxicity was evaluated by the rotarod neurotoxicity test. The results of these tests demonstrated that **147a** (Figure 25) and **147b** (Figure 25) were the most promising compounds, with median effective dose (ED_50_) of 17.6 and 17.9 mg/kg, and protective index (PI) of greater than 63.4 and 62.4 in the MES test, respectively. These PI values were higher than the PI value of the prototype antiepileptic drug carbamazepine. Sc-PTZ tests showed that **147a** was the most potent with ED_50_ value of 38.0 mg/kg and PI value of greater than 29.4, which is much safer than marketed drug carbamazepine^169^.

As a continuation of the work above, another series of 9-alkoxy-6,7-dihydro-2*H*-benzo[c][1,2,4]triazolo[4,3-a]azepin-3(5*H*)-one derivatives **148** (Figure 25) was designed and synthesised as anticonvulsant agents. In this work, compound **148a** (Figure 25) was obtained as the most active one with ED_50_ of 27.3 mg/kg, TD_50_ of 118.3 mg/kg, and PI value of 4.3. Generally, the activity was declining when compared to the prototype compounds **147**^170^.

Another series of triazolones containing phthalazine were prepared and evaluated for their anticonvulsant activity against MES-induced seizure. The result illustrated that **149** (Figure 25) possessed the most potential anticonvulsant activity with the 100% protection at 100 mg/kg in MES test^171^.

Next, a number of benzo[b][1,2,4]triazolo[4,3-d][1,4]-thiazin-1-one derivatives were synthesised as the bioisosterism of **144**, and tested for their anticonvulsant activity by MES test in mice. Based on the results of MES test and rotarod test, **150** (Figure 25) was considered as the most promising one, which showed an ED_50_ value of 9.2 mg/kg and a PI value of 15.4. The safety of compound **150** was superior to the standard drug carbamazepine^172^.

Following the above work, a series of triazolo[3,4-b]benzothiazol-3(2*H*)-ones was synthesised as the ring-reduce produces of **150**. In this series, the compound **151a** (Figure 25) and **151b** (Figure 25) showed high levels of anticonvulsant activity in MES test with ED_50_ of 11.4 and 13.6 mg/kg, respectively. It is worth mentioning that compound **151b** showed no neurotoxicity at 700 mg/kg, which led to a high protective index (PI > 51). In addition, the potency of compound **151b** against seizures induced by PTZ, 3-mercaptopropionic acid, and bicuculline suggested that it might exert its anticonvulsant activity through affecting the GABAergic system^173^.

A series of novel triazolones containing benzimidazole were synthesised and screened for their anticonvulsant activities. Among the synthesised compounds, **152** (Figure 25) was the most active compound with an ED_50_ of 31.69 mg/kg, a TD_50_ above 350 mg/kg, and PI value bigger than 11.04 by intraperitoneal administration in the mice. Meanwhile, compound **152** exhibited ED_50_ values of 44.01 mg/kg and the PI values of 31.6 by oral administration in mice^174^.

A series of 3,4-dihydroisoquinolin with triazolone derivatives were synthesised and evaluated for their anticonvulsant activity using MES test and PTZ induced seizure test. Among them, **153** (Figure 25) showed significant anticonvulsant activity in MES tests with an ED_50_ value of 63.31 mg/kg and it showed wide margins of safety with protective index (PI > 7.9). A docking study of compound **153** in the benzodiazepine (BZD)-binding site of aminobutyric acid A (GABAA) receptor confirmed possible binding of compound **153** with the BZD receptors^175^.

## 7. 1,2,3-Triazoles

**Figure 26.**
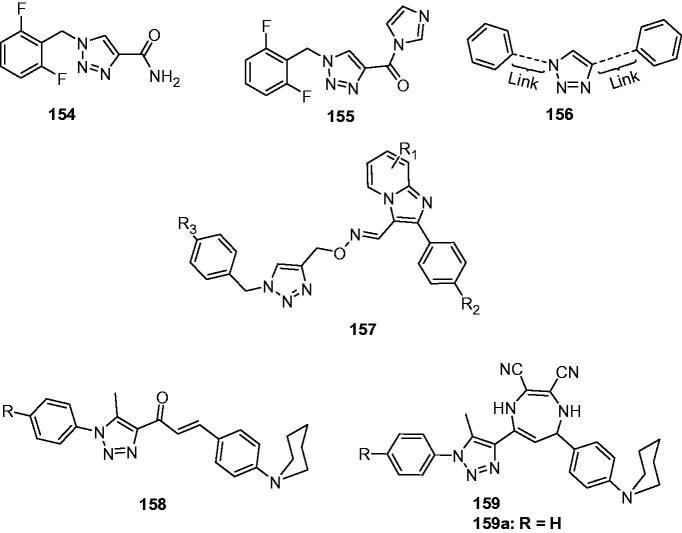
1,2,3-Triazoles with anticonvulsant activity (**154**–**159**).

Rufinamide (**154**, Figure 26), a 1,2,3-triazole derivative, was developed in 2004 and approved in 2008. It is frequently used to treat various seizure disorders, especially the seizures associated with Lennox–Gastaut syndrome in children 4 years and older and adults^176^. Based on the SAR of rufinamide, several 1-(2,6-difluorobenzyl)-1*H*-1,2,3-triazole were prepared by Shukla et al., and evaluated for CNS depressant and anticonvulsant activities by photoactometer, rotarod, and PTZ-induced convulsion method, respectively, in mice. The most promising one **155** exhibited the potent anticonvulsant activity in PTZ model with an ED_50_ of 5.5 mg/kg while the diazepam showed an ED_50_ of 1.0 mg/kg^177^.

It is well known that sodium channel is one of the important targets for antiepileptic drugs. Many marketed anticonvulsant agents are involved in the action mechanism of sodium channel inhibition. In 2012, a novel series of 1,4-disubstituted-[1,2,3]triazole derivatives (**156**, Figure 26) were prepared via copper-catalysed click chemistry as inhibition of NaV1.6 sodium channel currents. In electrophysiological evaluation, many of the molecules can block the rNav1.6 currents at 10 μM by over 20%, displaying IC_50_ values ranging from 19.6 to 125.4 μM. These founding will be exploited in the preparation of new compounds and could result in potentially useful AEDs^178^.

Ulloora et al. reported the synthesis and anticonvulsant studies of new 2-arylimidazo[1,2-a]pyridines containing 1,2,3-triazoles (**157**, Figure 26) as well as their intermediates. The anticonvulsant study was carried out by MES and scPTZ screening methods, while their toxicity study was performed by rotarod method. Some of these compounds exhibited complete protection against seizure at 20 mg/kg, which were comparable with standard drug diazepam. The c log*P* values of target compounds are in the range of 3.5–5.3, which confirm their lipophilic nature^179^.

Recently, a novel series of chalcone derivatives containing 1,2,3-triazole were synthesised via Claisen–Schmidt condensation reaction. All compounds **158** (Figure 26) and **159** (Figure 26) revealed anticonvulsant activity, and compounds **159** were found to be more active than compounds **158**. This activity may be attributed to the presence of the azepine nucleus in compounds **159** which has sedative like effect and increases the activity of these compounds as anticonvulsant, also the presence of the two cyano groups in compounds **159** makes the compounds more lipophilic than compounds **158**. The compound **159a** (Figure 26) was the most active one in this study with an ED_50_ of 25 and 28 mg/kg in the MES test and PTZ test, respectively^180^.

## Conclusion

In this paper, an attempt was made to systematically review the researches of triazole derivatives in the design and development of anticonvulsant agents in recent 20 years. From the review, we can see that big numbers of triazole derivatives were synthesised and identified with promising anticonvulsant activity. Their anticonvulsant activities were confirmed mainly but not limited to the *in vivo* screening methods. Their structures were focused on triazole moiety but abundant with various kinds of structures. From the presented review, it could be concluded that the triazole is an important fragment and has an affinity for some targets related to epilepsy-treatment. Many of triazole derivatives showed potent anticonvulsant activities with high selectivity and low toxicity, which can be used as lead compound to design and develop new anticonvulsant drugs. It seems that the role of triazole nucleus for designing anticonvulsants remains unclear, because it has no specifically target. But what does that matter? Because of the dimness of epilepsy pathogenesis and the complex mechanisms of the existing AEDs, the effectiveness and safety *in vivo* remain the most important factors in the discovery of new AEDs. Triazole nucleus plays different roles in these compounds for their anticonvulsants mediated by different molecular targets. Therefore, the introduction of triazole nucleus is relevant for designing newer compounds (new chemical entities) regardless of mechanism of action.
